# Generation and Application of Inducible Chimeric RNA *ASTN2-PAPPA*_as_ Knockin Mouse Model

**DOI:** 10.3390/cells11020277

**Published:** 2022-01-14

**Authors:** Yichen Luo, Liang Du, Zhimeng Yao, Fan Liu, Kai Li, Feifei Li, Jianlin Zhu, Robert P. Coppes, Dianzheng Zhang, Yunlong Pan, Shegan Gao, Hao Zhang

**Affiliations:** 1 Institute of Precision Cancer Medicine and Pathology, School of Medicine and Department of General Surgery, The First Affiliated Hospital of Jinan University, Jinan University, Guangzhou 510632, China; lyc0610@stu2020.jnu.edu.cn; 2 Department of Biomedical Sciences of Cells & Systems, Section Molecular Cell Biology and Radiation Oncology, University Medical Center Groningen, University of Groningen, 9700 AD Groningen, The Netherlands; l.du@umcg.nl (L.D.); r.p.coppes@umcg.nl (R.P.C.); 3 Graduate School, Shantou University Medical College, Shantou 515041, China; 4 Department of General Surgery, The First Affiliated Hospital of Jinan University, Jinan University, Guangzhou 510632, China; yaozhim-eng250@jnu.edu.cn (Z.Y.); qq123966766@stu2019.jnu.edu (J.Z.); tpanyl@jnu.edu.cn (Y.P.); 5 Institute of Precision Cancer Medicine and Pathology, School of Medicine, Jinan University, Guangzhou 510632, China; fanlgodwin@stu2020.jnu.edu.cn (F.L.); kail59@stu2019.jnu.edu.cn (K.L.); 6 Department of Oncology, People’s Hospital of Leshan, Leshan 614099, China; feifei_li112@163.com; 7 Department of Bio-Medical Sciences, Philadelphia College of Osteopathic Medicine, 4170 City Avenue, Philadelphia, PA 19131, USA; dianzhengzh@pcom.edu; 8 Henan Key Laboratory of Cancer Epigenetics, College of Clinical Medicine, The First Affiliated Hospital of Henan University of Science and Technology, Luoyang 471000, China; 9 Minister of Education Key Laboratory of Tumor Molecular Biology, Jinan University, Guangzhou 510632, China

**Keywords:** transcription-induced chimeric RNA, fusion RNA, *A-P*_as_chiRNA, genetically engineered mouse model, knockin mouse, cancer

## Abstract

Chimeric RNAs (chiRNAs) play many previously unrecognized roles in different diseases including cancer. They can not only be used as biomarkers for diagnosis and prognosis of various diseases but also serve as potential therapeutic targets. In order to better understand the roles of chiRNAs in pathogenesis, we inserted human sequences into mouse genome and established a knockin mouse model of the tamoxifen-inducible expression of *ASTN2-PAPPA* antisense chimeric RNA (*A-P*_as_chiRNA). Mice carrying the *A-P*_as_chiRNA knockin gene do not display any apparent abnormalities in growth, fertility, histological, hematopoietic, and biochemical indices. Using this model, we dissected the role of *A-P*_as_chiRNA in chemical carcinogen 4-nitroquinoline 1-oxide (4NQO)-induced carcinogenesis of esophageal squamous cell carcinoma (ESCC). To our knowledge, we are the first to generate a chiRNA knockin mouse model using the Cre-loxP system. The model could be used to explore the roles of chiRNA in pathogenesis and potential targeted therapies.

## 1. Introduction

Chimeric RNAs (chiRNAs) are known as fusion transcripts because they comprise RNA fragments derived from different genes [1,2]. Canonical chiRNAs are transcripts of chromosomally rearranged DNA, while non-canonical chiRNAs are formed via DNA-independent mechanism such as trans-splicing or transcription readthrough. With the development of advanced deep sequencing technologies, more non-canonical chiRNAs have been discovered [1,3,4]. Although the precise mechanisms underlying the formation of non-canonical chiRNA have not been completely elucidated [5,6], accumulating evidence indicates that chiRNAs play important roles in a broad spectrum of diseases, including cancer [2,7]. Given their unique expression in the tissues/cells, a subgroup of chiRNAs can serve not only biomarkers for both diagnosis and prognosis [5,8] but also potential therapeutic targets [9].

We have previously investigated the potentials of chiRNAs as biomarkers and molecular targets in cancer [10,11,12]. We examined salivary exosomal *GOLM1-NAA35* chiRNA (se*G-N*chiRNA) in patients with esophageal squamous cell carcinoma (ESCC) and found that se*G-N*chiRNA can serve as a convenient, reliable, and noninvasive biomarker for the assessment of therapeutic response and recurrence [12]. In addition, *ASTN2-PAPPA*_antisense_ chimeric RNA (*A-P*_as_chiRNA), which is generated by transcription read-through/splicing or trans-splicing, is also highly expressed in ESCC but not the surrounding normal esophagus. Results derived from xenograft models indicate that *A-P*_as_chiRNA can promote ESCC cell spread to lymph nodes and enhance stemness through modulating OCT4. Mechanistically, *A-P*_as_chiRNA induces cancer stemness by activating extracellular-signal-regulated kinase 5 (ERK5)-mediated non-canonical RNA Polymerase II-Associated Factor 1 Homolog (PAF1) [11]. These findings suggest that chiRNAs could play critical roles in cancer pathogenesis. In order to better understand the role of *A-P*_as_chiRNA in pathogenesis as well as the underlying molecular mechanisms, we decided to establish a mouse model conditionally expressing *A-P*_as_chiRNA. 

The Cre-loxP-mediated recombination system has been widely used to create genetically engineered mouse models [13]. In addition, tamoxifen-induced gene expression has been proven to be helpful for the control of conditional expressions [14,15,16,17]. In this study, we first generated and characterized a tamoxifen-inducible *A-P*_as_chiRNA knockin (KI) mouse model and then induced ESCC by 4-nitroquinoline 1-oxide (4NQO). By controlling its expression, we demonstrated the impact of *A-P*_as_chiRNA on ESCC development. 

## 2. Materials and Methods

### 2.1. Establishment and Characterization of A-P_as_chiRNA Conditional Knockin Model

Mice used in this study were generated from crossing *A-P*_as_chiRNA*^flox/flox^* mice with CAG-CreER mice on C57BL/6 background. CAG-CreER mice were originally generated by Jackson Model Animal Laboratory and provided by Cyagen Biosciences (Guangzhou, China). The “CAG promoter-loxP-3*polyA-loxP-*A-P*_as_ sequence-polyA” cassette was inserted into intron 1 of ROSA26. The targeting vector was obtained from Cyagen Biosciences (Guangzhou, China). In the targeting vector, the Neo (neomycin resistance gene) cassette was flanked by SDA (self-deletion anchor) sites, and DTA (diphtheria toxin A) was used for negative selection. Correctly inserted constructs were confirmed by digestions with different restriction enzymes and DNA sequencing (Supplementary Table S1). 3-month-old male and female mice were administered tamoxifen (Cat. T-5648, Sigma, St. Louis, MO, USA) for 5 consecutive days (50 mg/kg body weight; intraperitoneal injection). Mice were genotyped for floxed and Cre alleles as well as the excised allele after tamoxifen induction using the following primers: F4 (5′-GGAAAGAACCAGCTGGGGGATATC-3′), R4 (5′-GCCATTTAAGCCATGGGAAGTTAG-3′), and F5 (5′-TGGACAGAGGAGCCATAACTGCAG-3′) for targeted allele; Primers F1 (5′-CATATTGGCAGAACGAAAACGC-3′) and R1 (5′-CCTGTTTCACTATCCAGGTTACGG-3′) for Cre transgene. PCR products were separated on a 2% agarose gel to verify the DNA size [18]. All mice were housed under strictly controlled daily lighting (12 h light/dark) at 20–22 °C and 50–55% humidity and were provided ultra-filtered water and food ad libitum. All animal procedures were approved by the Animal Care and Use Committee of Jinan University (IACUC-20191210-03). 

### 2.2. Targeting A-P_as_chiRNA to Embryonic Stem Cells (ES Cells)

The ROSA26 targeting construct was linearized by restriction enzyme NotI (R3189L, New England Biolabs, Ipswich, MA, USA), followed by phenol/chloroform extraction and ethanol precipitation. The linearized vector was transfected into C57BL/6 ES cells by electroporation, and the transfected ES cells were subject to G418 selection (200 μg/mL) for 24 h. The following primers were used for screening potentially targeted clones: primers P3 (5′-CAAAGCTGAAAGCTAAGTCTGCAG-3′) and P4 (5′-GGGCCATTTACCGTAAGTTATGTAACG-3′) for KI1 PCR; primers P5 (5′-CAACGTGCTGGTTATTGTGCTGTCT-3′) and P6 (5′-TGGTGGCCACGTGTAGTGGATCC-3′) for KI2 PCR. The positive clones (1A1, 1A8, 1A10, 1E3, 1G3, and 1H2) were expanded and further characterized by Southern blot analysis (Supplementary Figure S2F). DNAs were digested by endonucleases, separated by electrophoresis, transferred to a nitrocellulose membrane, and hybridized with labeled DNA probes. All six expanded clones were confirmed to be correctly targeted.

### 2.3. Generation of Mice Expressing chiRNA and Breeding Scheme

Targeted ES cell clone 1E3 was injected into C57BL/6 albino embryos, which were then re-implanted into CD-1 pseudo-pregnant females. Founder animals were identified by their coat color, and their germline transmission was confirmed by breeding with C57BL/6 females and subsequent genotyping of the offspring. The Neo cassette is self-deleted in germ cells so the offsprings were Neo cassette-free. F1 offspring were identified by PCR for the presence of the *A-P*_as_*^flox/+^* allele using genomic DNA isolated from tail tissue. The following primers were used for screening for KI1, KI2, KI3, and Neo deletion: F1 (5′-CATATTGGCAGAACGAAAACGC-3′) and R1 (5′-CCTGTTTCACTATCCAGGTTACGG-3′) for KI1; F2 (5′-GCATCCTCAAGGACACCAAAATCAC-3′) and R2 (5′-GATATCCCCCAGCTGGTTCTTTCC-3′) for KI2; F3 (5′-CAACGTGCTGGTTATTGTGCTGTCT-3′) and R3 (5′-TGGTGGCCACGTGTAGTGGATCC-3′) for KI3; and F4 (5′-GGAAAGAACCAGCTGGGGGATATC-3′), R4 (5′-GCCATTTAAGCCATGGGAAGTTAG-3′), and F5 (5′-TGGACAGAGGAGCCATAACTGCAG-3′) for neo deletion (Supplementary Figure S3A). *A-P*_as_*^flox/flox, CAG-Cre^* mice were generated via crossing *A-P*_as_*^flox/+^* mice with CAG-CreER mice. After tamoxifen induction, *A-P*_as_chiRNA expressions in vital organs (liver, intestines, ovary, heart, brain, muscle, kidney, skin, tongue, stomach, lung, spleen, and esophagus) were detected by real-time quantitative PCR (RT-qPCR) [18]. The experiment process of RT-qPCR is described as follows. Total RNA was isolated from mice tissues by the TRIzol reagent (Cat. 15596-018, ThermoFisher, Waltham, MA, USA) and reverse transcribed using a High Capacity cDNA Reverse Transcription Kit (Cat. 4368813, Applied Biosystems, Waltham, MA, USA) according to the manufacturer’s instructions. cDNA was amplified and quantified in the CFX Connect system (Cat. 1855200, BIO-RAD, Hercules, CA, USA) by using SYBR Green Master (Cat. 08733/09121, CWBIO, China). Reactions were performed using a total volume of 20 μL, which contained 1 μL of cDNA template (corresponding to 5 ng of the starting amount of RNA), 0.2 mM each primer, and 10 μL 2× SYBR Premix Ex Taq II. PCR cycling conditions were as follows: 94 °C for 30 s, followed by 40 cycles of 94 °C for 10 s, 55–62 °C for 10 s, and 72 °C for 10 s in a 96-well reaction plate, and the annealing temperature was based on the Tm value of primers. The melting curve was recorded after 40 cycles to verify primer specificity by heating from 65 °C to 95 °C. Each RT-qPCR reaction was performed in triplicate (technical replicates) on samples from three individual plants (biological replicates). cDNA was subjected to RT-qPCR with the following primers: *A-P*_as_chiRNA forward: 5′-CAACGTGCTGGTTATTGTGCTGTCT-3′ and reverse: 5′-TGGTGGCCACGTGTAGTGGATCC-3′; β-actin forward: 5′-GAACCCCAAGGCCAACCGCGAGA-3′ and reverse: 5′-TGACCCCGTCACCGGAGTCCATC-3′. β-actin served as a reference gene for normalization.

### 2.4. Induction the Expression of ESCC Model by 4NQO with or without Expression of chiRNA

Wild type (WT) and *A-P*_as_chiRNA KI mice (3 males and 3 females per group) weighing 20–22 g were randomly selected 4 weeks after the tamoxifen injection and anesthetized with 50 mg/kg body weight of avertin (2, 2, 2, -Tribromoethanol, 20 mg/mL, Sigma, St. Louis, MO, USA). Blood was collected from the canthal vein and subjected to cytological and biochemical tests. Small pieces of liver, kidney, spleen, lung, heart, and brain were fixed in formalin for 24 h and embedded in paraffin. After deparaffinization in xylene, the tissue sections (5 μm) were stained with Hematoxylin and Eosin (HE) and examined by an experienced veterinary pathologist. Six-week-old WT and *A-P*_as_chiRNA KI mice treated with tamoxifen were given 100 μg/mL carcinogen 4NQO (Cat. N8141, Sigma, St. Louis, MO, USA) in drinking water for 16 weeks [19]. Whole esophagi were longitudinally dissected and pictured. The gross lesions (>1 mm diameter) of esophagi were counted and fixed in formalin. The bodyweight of the mice was measured weekly.

### 2.5. Statistical Analysis

Student’s *t*-test or analysis of variance (ANOVA) followed by appropriate multiple comparison tests using SPSS statistics 20.0 software. When *p* < 0.05, it is considered statistically significant.

## 3. Results

### 3.1. Construction of the Targeting Vector

Rosa26 (NCBI Reference Sequence: NR_027008.1) is the most commonly used “safe harbor” locus because Rosa26 encodes a nonessential nuclear RNA expressed in almost all tissues. The conditional expression of an exogenous gene will result when a loxP-3*stop-loxP sequence is inserted upstream of the exogenous sequence at the Rosa26 locus, and this model was crossed with a Cre deleter. As illustrated in Supplementary Figure S1A, we inserted the “CAG promoter-loxP-3*polyA-loxP-*A-P*_as_ sequence-polyA” cassette into intron 1 of ROSA26 to build gene-edited mouse models. We used high-fidelity Taq DNA polymerase to amplify mouse genomic fragments containing homology arms (Has) from BAC (bacterial artificial chromosome) clone and assembled into a targeting vector together with recombination sites and selection markers (Supplementary Figure S1B). Next, the targeting vector was linearized with restriction enzymes and used as a PCR template. Supplementary Figure S1C showed expected PCR bands indicating that the targeting clone had been successfully constructed.

### 3.2. Generation and Screening of ES Cells

The ROSA26 targeting construct was linearized by restriction enzyme NotI, followed by phenol/chloroform extraction and ethanol precipitation. Linearized DNA was transfected into C57BL/6 ES cells by electroporation, and transfected ES cells were subject to G418 selection (200 μg/mL) 24 h post electroporation. One-hundred and eighty-six G418-resistant clones were amplified in 96-well plates. Two 96-well plates were made, with one of them frozen at −80 °C and the other was used for DNA isolation and subsequence PCR screening for homologous recombination. The PCR screening identified 37 potential clones (Supplementary Figure S2A–D). The positive clones (1A1, 1A8, 1A10, 1E3, 1G3, and 1H2) were expanded and further characterized by Southern blot analyses. DNA was digested with either EcoNI or EcoRV and hybridized using a Neo probe, which was expected to detect the following DNA fragment from the targeted allele: ~15.13 kb (with EcoNI digestion) and ~11.01 kb (with EcoRV digestion) (Supplementary Figure S2E,F). These results suggest all clones are correctly targeted.

### 3.3. Genotyping of F1 A-P_as_chiRNA^flox/+^ Mice

Targeted ES cell clone 1E3 was injected into C57BL/6 albino embryos and re-implanted into CD-1 pseudo-pregnant females. Founder animals were identified by their coat color. Their germline transmission was confirmed by breeding with C57BL/6 females and subsequent genotyping of the offspring. The Neo cassette is self-deleted in germ cells; thus, the offspring is Neo cassette-free. As shown in Supplementary Figure S3A, KI1 (F1 and R1), KI2 (F2 and R2), KI3 (F3 and R3), and Neo (F4 and R4 and F5 and R4) were confirmed with the insertion of fragment pairs and Neo self-removal. Finally, three male and seven female heterozygous mice (F1 generation: #1, #8, #9, #10, #12, #15, #18, #20, #21, and #22) were identified as positive KI1 and KI2 (Supplementary Figure S3B,C). The *A-P*_as_chiRNA positive pups were reconfirmed by PCR screening for KI3 and Neo deletion (Supplementary Figure S3D,E).

### 3.4. Tamoxifen-Inducible Expression of A-P_as_chiRNA

**Figure 1:**
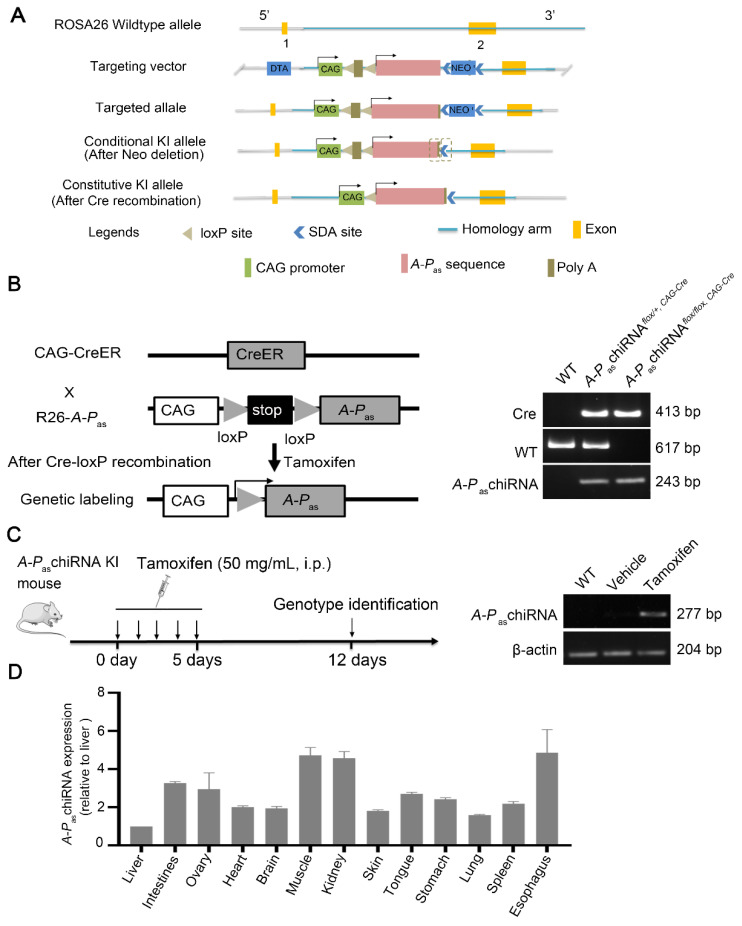
Genotyping of the *A-P*_as_chiRNA *^flox/flox, CAG-Cre^* mice. (**A**) Schema of structure of *A-P*_as_chiRNA KI mice. (**B**) Scheme for genetic lineage tracing strategy (left panel). Wild type (WT), heterozygous (hetero), and homozygous (homo) mice were clearly distinguished according to Supplementary Figure S3A (right panel). Wild type: 617 bp (using primer F5/R4), *A-P*_as_chiRNA homozygotes: 243 bp (using primer F4/R4), *A-P*_as_chiRNA heterozygotes: 243 bp/617 bp (using primer F4/R4 and F5/R4), and Cre amplicon: 413 bp (using primer F1/R1). (**C**) Experimental strategy for tamoxifen induction (left panel). *A-P*_as_chiRNA expression was detected by PCR after tamoxifen induction. The PCR product of *A-P*_as_chiRNA was 277 bp, (using primer F3/R3) (right panel). (**D**) Quantification of the expression of *A-P*_as_chiRNA in different organs. *A-P*_as_chiRNA was highly enriched in the adult vital organs. Quantification of the expression of *A-P*_as_chiRNA in different organs of the *A-P*_as_chiRNA*^flox/flox, CAG-Cre^* mice after tamoxifen induction. *A-P*_as_chiRNAs were normalized to β-actin (ΔCt = Ct (*A-P*_as_chiRNA) − Ct (β-actin)). The results are shown as average fold change relative to liver, the lowest expression of *A-P*_as_chiRNA in organ, using 2^−ΔΔCt^, which ΔΔCt = ΔCt (organ) − ΔCt (liver).

The schema of structure of *A-P*_as_chiRNA KI mice was shown in Figure 1A. We then crossed CAG-CreER with a responsive reporter line, R26-*A-P*_as_chiRNA, to generate the *A-P*_as_chiRNA*^flox/flox, CAG-Cre^* mouse and genotyped by PCR (Figure 1B). The tamoxifen induction strategy is shown in Figure 1C (left panel). The genotypes of mice induced by tamoxifen or vehicle are shown in Figure 1C (right panel). In order to examine *A-P*_as_chiRNA expression in adult tissues, we treated 8-week-old to 10-week-old *A-P*_as_chiRNA*^flox/flox, CAG-Cre^* mice with tamoxifen for 5 days before tissue samples were collected. *A-P*_as_chiRNA expression in the vital organs including heart, brain, lung, liver, intestine, stomach, kidney, spleen, ovary, tongue, esophagus, muscle, and skin were significantly increased when *A-P*_as_chiRNA*^flox/flox, CAG-Cre^* mice were treated with tamoxifen (Figure 1D). Before tamoxifen induction, the expression of *A-P*_as_chiRNA could not be detected by RT-qPCR in multiple organs of mice (Supplementary Figure S4A). These data altogether suggest that we have successfully established a KI mouse model with inducible expression of *A-P*_as_chiRNA.

### 3.5. Evaluation of the Phenotype Characterization of A-P_as_chiRNA KI Mice

**Figure 2:**
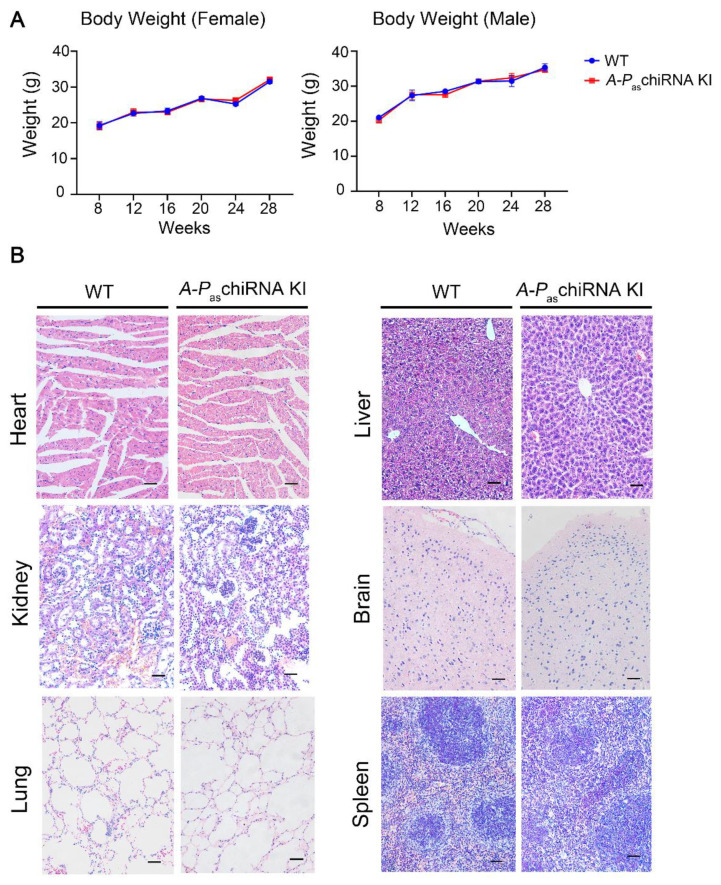
Body weight and major organs in WT and *A-P*_as_chiRNA KI mice. (**A**) Body weight of WT and *A-P*_as_chiRNA KI mice was measured weekly for 20 weeks. Body weight of female (n = 3 per group; left panel) and male mice (n = 3 per group; right panel) did not differ significantly between two groups. (**B**) HE staining of major organs (i.e., heart, kidney, lung, liver, brain, and spleen). Scale bar: 50 μm.

No significant differences between the WT and *A-P_as_*chiRNA KI mice were observed in body weight, fur, and other physiological indices after 8 weeks of tamoxifen treatment (Figure 2A). The fertility of female *A-P*_as_chiRNA KI mice was not affected by the expression of tamoxifen-induced *A-P*_as_chiRNA since there were no obvious changes in the average number of pregnancies, litter size, and morbidity and mortality in *A-P*_as_chiRNA KI mice (Supplementary Table S2). In addition, eight physiological indexes and nine biochemical indexes were also measured. Hematology results indicated that all measured factors were within normal ranges, suggesting that tamoxifen-induced *A-P*_as_chiRNA did not induce inflammatory responses (Supplementary Table S3). There was no significant difference in liver and kidney function indexes between the two groups (Supplementary Table S4). In addition, HE staining of the major organs including brain, heart, liver, spleen, lung, and kidney (Figure 2B) showed no significant difference between *A-P*_as_chiRNA KI and WT mice. These data altogether indicate that tamoxifen-induced *A-P*_as_chiRNA does not affect the general physiology of the mice.

### 3.6. Chemical Carcinogen-Induced A-P_as_chiRNA KI Mice Mimicking Human ESCC

**Figure 3:**
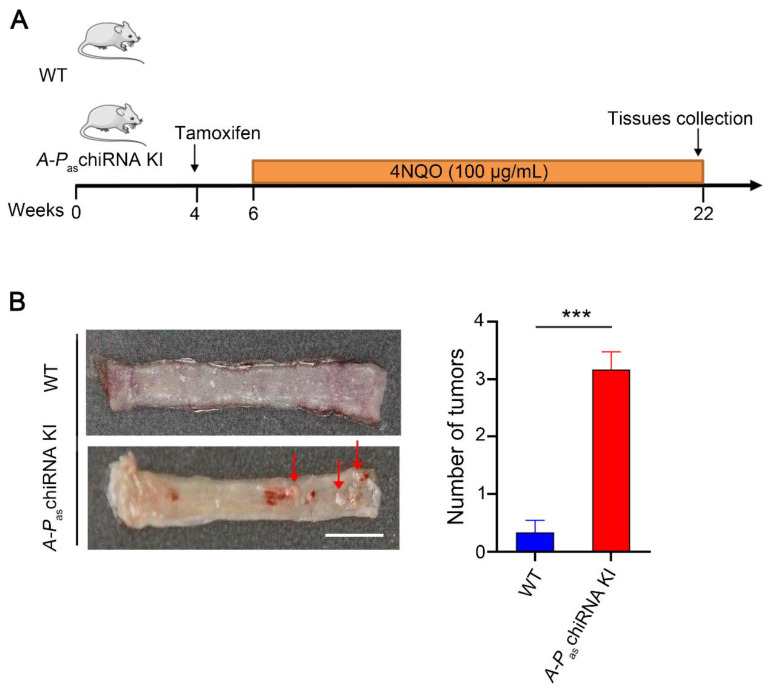
4NQO-induced ESCC tumors in WT and *A-P*_as_chiRNA KI mice. (**A**) The protocol of chemical-induction of ESCC in WT and *A-P*_as_chiRNA KI mice. Mice were given 4NQO (100 μg/mL) in drinking water for 16 weeks. (**B**) The left panel demonstrates the gross anatomy of the representative esophagus from WT and *A-P*_as_chiRNA KI mice. Red arrows indicate esophageal tumors. The number of tumors per mouse in the esophagus from WT and *A-P*_as_chiRNA KI mice was shown in the right panel. n = 6 per group; *** *p* < 0.001; Scale bar: 5 mm.

Next, we examined the role of *A-P*_as_chiRNA in the carcinogenesis of ESCC when mice were exposed to carcinogen 4NQO (Figure 3A). The mice drank water containing 4NQO (100 μg/mL) for 16 weeks before esophageal tissues were collected. Figure 3B showed that *A-P*_as_chiRNA dramatically increased 4NQO-induced ESCC initiation (the number of tumors) compared with WT mice. As previously described, the esophageal wall of WT mice was thick, rough, and lost proper organization at the end of the 16-week 4NQO treatment [20,21]. However, with identical treatment, the esophagi form mice expressing *A-P*_as_chiRNA deteriorated rapidly and exhibited multiple visible lesions (>1 mm diameter). *A-P*_as_chiRNA KI mice prior to tamoxifen induction were also fed with 4NQO in drinking water to observe tumor formation during this experiment, and it was found that there was no significant difference in tumor formation between WT mice and *A-P*_as_chiRNA KI mice untreated with tamoxifen (Supplementary Figure S4B). HE staining of the esophagus of WT and *A-P*_as_chiRNA KI mice after 4NQO treatment. The results showed that the esophageal wall of WT mice had a loss of organization of the epithelium (dysplasia), whereas *A-P*_as_chiRNA KI mice exhibited the formation of esophageal squamous cell carcinoma and the invasion of neoplastic epithelial cells into subepithelial tissues (invasive carcinoma) (Supplementary Figure S4C). Altogether, these results indicate that *A-P*_as_chiRNA is capable of enhancing 4NQO-induced tumorigenesis and driving cancer cells to be more aggressive.

## 4. Discussion

Discovery of chiRNAs has added a novel layer of complex RNA universe and provided insights into molecular mechanisms of human diseases. Results from numerous studies suggest that chiRNAs not only play important roles in cancer pathogenesis but also can serve as biomarkers and therapeutic targets [10,11,12,22,23,24,25]. Different tools tailored explicitly to chiRNA research have been developed [7]. However, applicable in vivo mouse models for studying chiRNA in physiological state are lacking. Using the Cre-loxP system, we established an in vivo model to control the expression of *A-P*_as_chiRNA by tamoxifen in C57BL/6 mice with a few advantages. In fact, *A-P*_as_chiRNA KI mice are born without any embryonic lethal, and there is no obvious pathological change in organs nor abnormal metabolism. Since *A-P*_as_chiRNA can be expressed at the desired time point, this model can be used in different experiments to address specific scientific questions that other available models could not answer. As shown in this study, controlling the expression of chiRNA enabled us to dissect the role of *A-P*_as_chiRNA in 4NQO-induced ESCC. Additionally, since controlled expression of *A-P*_as_chiRNA can occur in many organs other than the esophagus including brain, heart, lungs, liver, stomach, colon, and spleen tissues, this model can broaden our understanding of *A-P*_as_chiRNA in physiological and pathological settings.

Without the tamoxifen-inducible *A-P*_as_chiRNA KI mouse model, we were able to show that *A-P*_as_chiRNA is highly enriched in ESCC [11], while whether the enriched expression of *A-P*_as_chiRNA is the causation or resultant of ESCC pathogenesis is still unknown. The inducible model enabled us to show the role of *A-P*_as_chiRNA in 4NQO-induced ESCC initiation, evidenced by the increased number of the tumor compared with WT mice. Previously, we demonstrated that *A-P*_as_chiRNA can induce cancer stemness by activating ERK5-mediated non-canonical PAF1 [11]. With this inducible model, we further showed the enhancing effect of *A-P*_as_chiRNA on tumor progression because cancer cells become more malignant in the presence of *A-P*_as_chiRNA. These findings not only support our previous reports but enable us to understand the underlying molecular mechanism at different levels. 

One limitation of our study was that the expression of *A-P*_as_chiRNA induced by tamoxifen occurs in multiple organs, we cannot exclude the possible effect from overexpressed *A-P*_as_chiRNA in the other organs. Nevertheless, studies using this animal model with a combination of different research tools will investigate unique questions that otherwise not be answered. To our knowledge, we are the first to establish a chiRNA KI mouse model. The methodology used in this report will be valuable for establishing knockin animal models of other chiRNAs.

## Data Availability

The data presented in this study will be made available upon reasonable request.

## Supplementary Materials

The following supporting information can be downloaded at: https://www.mdpi.com/article/10.3390/cells11020277/s1, Table S1: Sequence of the final targeting vector, Table S2: The fertility of two groups of female mice, Table S3: Hematological counts of two groups of mice, Table S4: Serum biochemical indices of two groups of mice. Figure S1: Experimental design for generation of targeting vector, Figure S2: Characterization of the ES clones, Figure S3: Genotype identification of F1 A-PaschiRNA flox/+ mice, Figure S4: The characteristic of A-PaschiRNA in mice.
