# Supplementary material for: Generation and Application of Inducible Chimeric RNA ASTN2-PAPPAas Knockin Mouse Model
Source: Cells. 2022 Jan 14;11(2):277. doi: 10.3390/cells11020277 (PMC8773765; doi:10.3390/cells11020277)
Supplement: Supplementary file 1 [file cells-11-00277-s001.zip › cells-1465623-supplementary/Supplementary Figures .pdf]

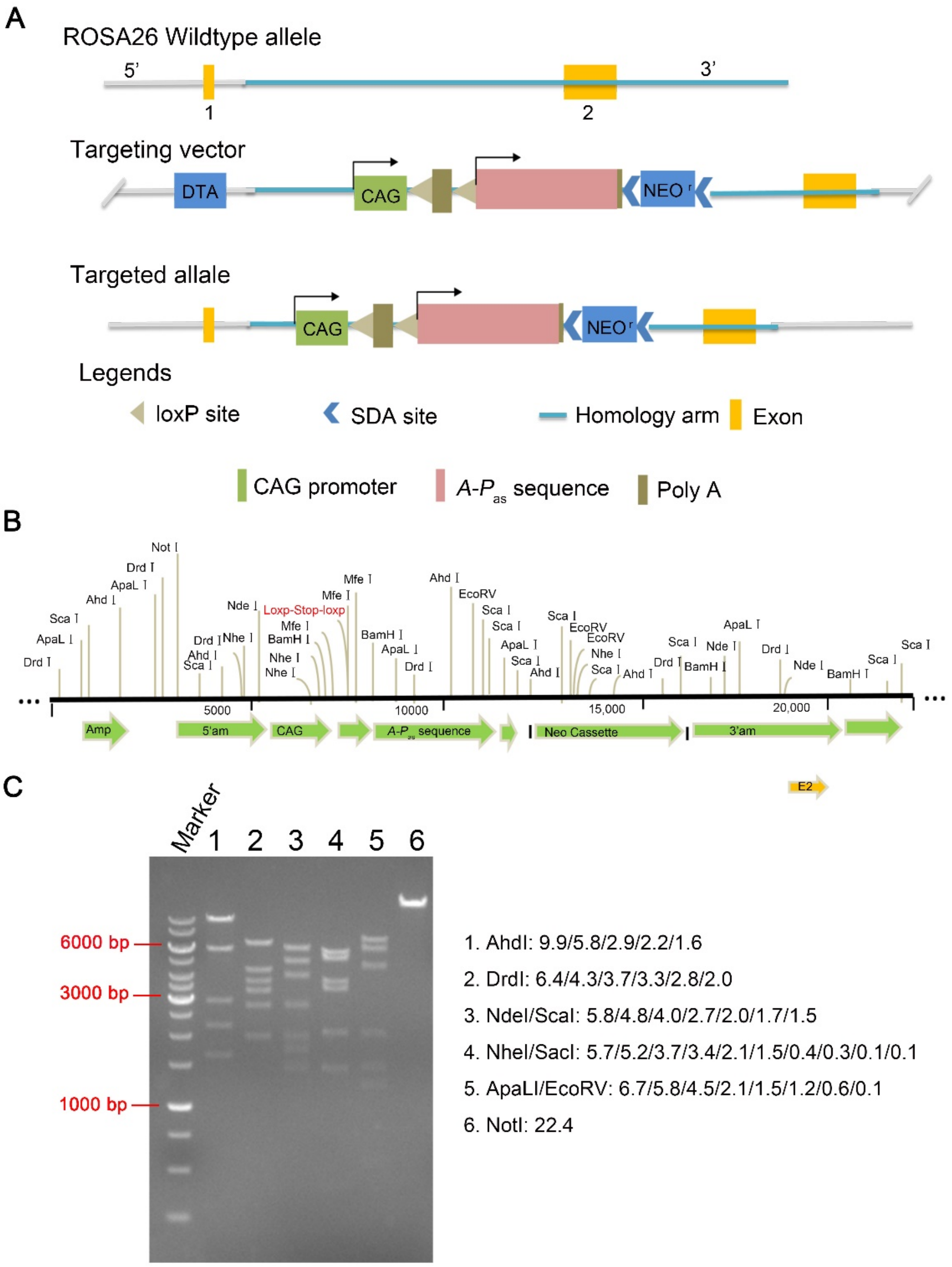

Supplementary Figure S1. Experimental design for generation of targeting vector. **(A)** The targeting vector and *A-P*$_{as}$^{floxneo} targeted allele. The ROSA26 gene is located on mouse chromosome 6. In the targeting vector, the positive selection marker (Neo) was flanked by SDA (self-deletion anchor) site. DTA was used for negative selection. **(B)** Diagram shows linearized targeting vector after restriction enzyme Not I linearization. Homology arms (HAs) were comprised of DTA cassette, 5′arm, Neo cassette, 3′arm, and Amp. **(C)** Verification of the final construct digested with Not I and separated on agarose gel.

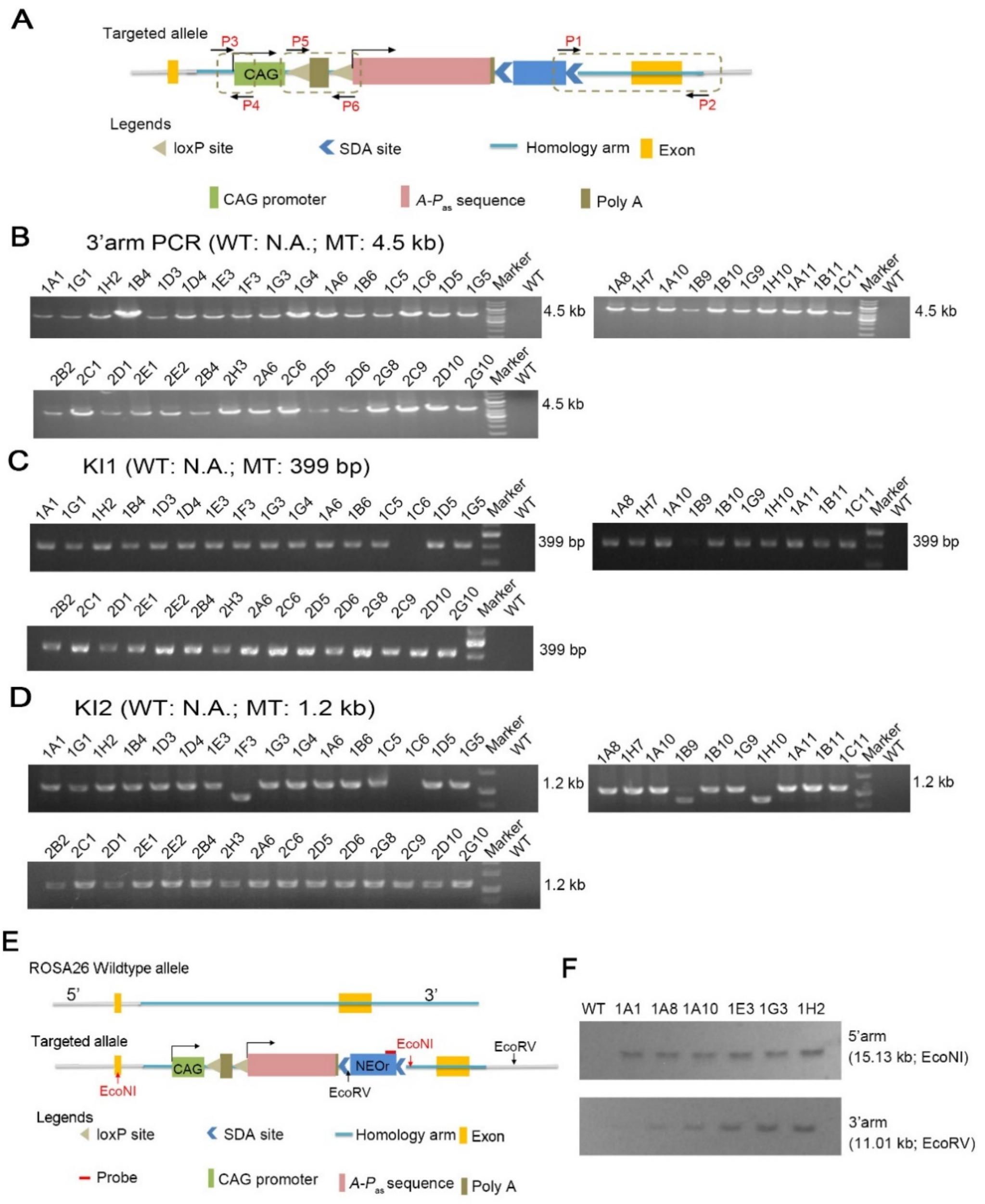

Supplementary Figure S2. Characterization of the ES clones. **(A)** Regions in the targeting vector were selected for PCR screening. **(B)** The potentially targeted clones were screened by 3'arm PCR (using primer P1/P2). **(C-D)** The potentially targeted clones were further screened by PCR for the presence of the distal KI1 site (using primer P3/P4) **(C)** and the KI2 site (using primer P5/P6) **(D)**. Samples 1A1, 1G1, 1H2, 1B4, 1D3, 1D4, 1E3, 1G3, 1G4, 1A6, 1B6, 1C5, 1D5, 1G5, 1A8, 1H7, 1A10, 1B10, 1G9, 1A11, 1B11, 1C11, 2B2, 2C1, 2D1, 2E1, 2E2, 2B4, 2H3, 2A6, 2C6, 2D5, 2D6, 2G8, 2C9, 2D10 and 2G10 have been confirmed as potentially targeted ES clones. **(E)** The positive clones from PCR screening were expanded and further characterized by Southern blot analysis. The EcoNI and EcoRV regions in the diagram were selected for Southern strategy. **(F)** Targeted clones (1A1, 1A8, 1A10, 1E3, 1G3 and 1H2) were subjected to Southern analysis of EcoNI digested genomic DNA with a Neo probe (5'arm 15.13 kb; upper panel) and EcoRV digested genomic DNA with a Neo probe (3'arm 11.01 kb; bottom panel). All of the six ES clones were confirmed correct by Southern blot analysis.

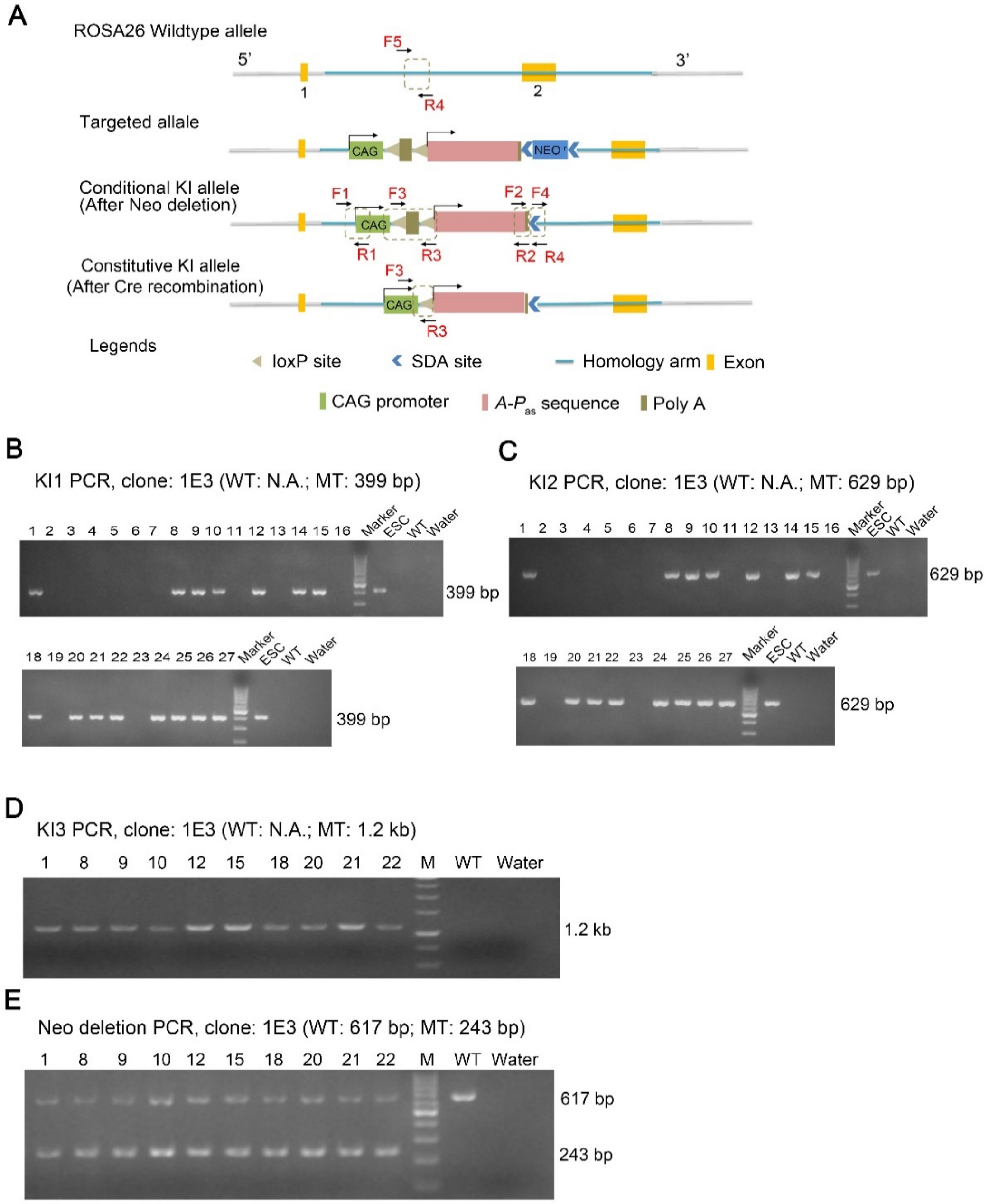

Supplementary Figure S3. Genotype identification of F1 *A-P*$_{as}$chiRNA $^{flox/+}$ mice. **(A)** Strategy for identifying the F1 *A-P*$_{as}$chiRNA $^{flox/+}$ mouse genotype by PCR screening for dashed line box regions (using primer F1, R1, F2, R2, F3, R3, F4, R4, F5, and R5). **(B-C)** Fifteen mice (#1, #8, #9, #10, #12, #14, #15, #18, #20, #21, #22, #24, #25, #26, and #27) from clone1E3 were identified as positive by PCR screening for KI1 (using primer F1/R1) **(B)** and KI2 (using primer F2/R2) **(C)**. **(D-E)** The positive pups (#1, #8, #9, #10, #12, #15, #18, #20, #21, and #22) were reconfirmed by PCR screening for KI3 (using primer F3/R3) **(D)** and Neo deletion (using primer F4/R4 and F5/R4) **(E)**.

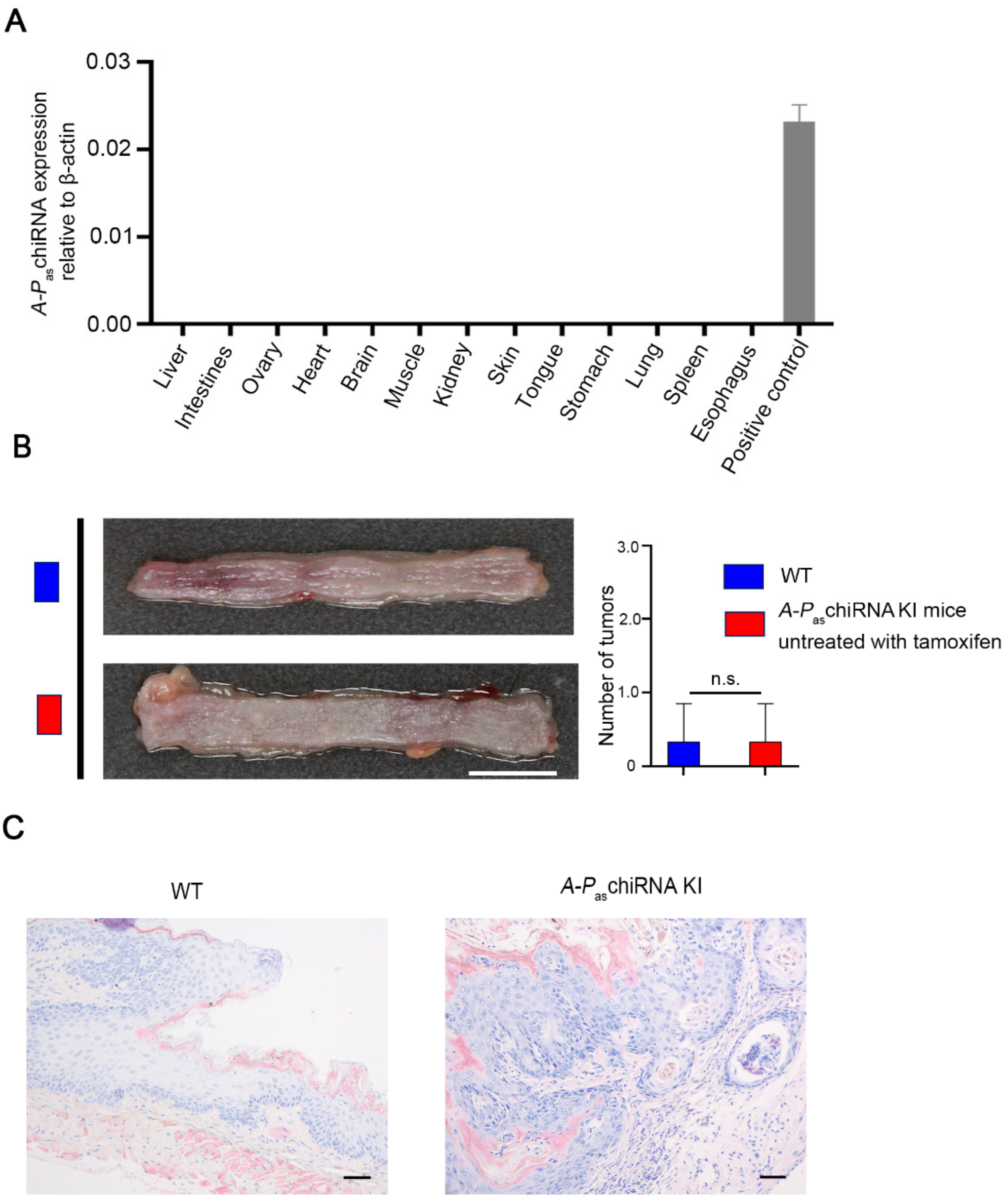

Supplementary Figure S4. The characteristic of *A-P*$_{as}$chiRNA in mice. **(A)** Quantification of the expression of *A-P*$_{as}$chiRNA in different organs of the *A-P*$_{as}$chiRNA KI mice before tamoxifen induction. The esophagus of mice after Tamoxifen induction were used as positive control. *A-P*$_{as}$chiRNA expression calculated as 2-ΔCt relative to β-actin. **(B)** The gross anatomy of the representative esophagus from WT mice and *A-P*$_{as}$chiRNA KI mice untreated with tamoxifen (left panel). The number of tumors per mouse in the esophagus from WT mice and *A-P*$_{as}$chiRNA KI mice untreated with tamoxifen (right panel). n = 6 pre group; Scale bar: 5 mm. n.s., not statistically significant; **(C)** The hematoxylin and eosin stain of the esophagus of WT mice and *A-P*$_{as}$chiRNA KI mice treated with tamoxifen after 4NQO treatment. Scale bar: 50 μm.
