# Supplementary material for: Generation and Application of Inducible Chimeric RNA ASTN2-PAPPAas Knockin Mouse Model
Source: Cells. 2022 Jan 14;11(2):277. doi: 10.3390/cells11020277 (PMC8773765; doi:10.3390/cells11020277)
Supplement: Supplementary file 1 [file cells-11-00277-s001.zip › cells-1465623-supplementary/Supplementary Table S1.pdf]

Supplementary Table S1. Sequence of the final targeting vector

1 TTAATA TTTTGT TAAAAT TCGCGT TAAATT TTTGTT AAATCA GCTCAT TTTTTA ACCAAT

61 AGGCCG AAATCG GCAAAA TCCCTT ATAAAT CAAAAG AATAGA CCGAGA TAGGGT TGAGTG

121 TTGTTC CAGTTT GGAACA AGAGTC CACTAT TAAAGA ACGTGG ACTCCA ACGTCA AAGGGC

181 GAAAAA CCGTCT ATCAGG GCGATG GCCCAC TACGTG AACCAT CACCCT AATCAA GTTTTT

241 TGGGGT CGAGGT GCCGTA AAGCAC TAAATC GGAACC CTAAAG GGAGCC CCCGAT TTAGAG

301 CTTGAC GGGGAA AGCCGG CGAACG TGGCGA GAAAGG AAGGGA AGAAAG CGAAAG GAGCGG

361 GCGCTA GGGCGC TGGCAA GTGTAG CGGTCA CGCTGC GCGTAA CCACCA CACCCG CCGCGC

421 TTAATG CGCCGC TACAGG GCGCGT CAGGTG GCACTT TTCGGG GAAATG TGCGCG GAACCC

481 CTATTT GTTTAT TTTTCT AAATAC ATTCAA ATATGT ATCCGC TCATGA GACAAT AACCCT

541 GATAAA TGCTTC AATAAT ATTGAA AAAGGA AGAGTA TGAGTA TTCAAC ATTTCC GTGTCG

601 CCCTTA TTCCCT TTTTTG CGGCAT TTTGCC TTCCTG TTTTTG CTCACC CAGAAA CGCTGG

661 TGAAAG TAAAAG ATGCTG AAGATC AGTTGG GTGCAC GAGTGG GTTACA TCGAAC TGGATC

721 TCAACA GCGGTA AGATCC TTGAGA GTTTTC GCCCCG AAGAAC GTTTTC CAATGA TGAGCA

781 CTTTTA AAGTTC TGCTAT GTGGCG CGGTAT TATCCC GTATTG ACGCCG GGCAAG AGCAAC

841 TCGGTC GCCGCA TACACT ATTCTC AGAATG ACTTGG TTGAGT ACTCAC CAGTCA CAGAAA

901 AGCATC TTACGG ATGGCA TGACAG TAAGAG AATTAT GCAGTG CTGCCA TAACCA TGAGTG

961 ATAACA CTGCGG CCAACT TACTTC TGACAA CGATCG GAGGAC CGAAGG AGCTAA CCGCTT

1021 TTTTGC ACAACA TGGGGG ATCATG TAACTC GCCTTG ATCGTT GGGAAC CGGAGC TGAATG

1081 AAGCCA TACCAA ACGACG AGCGTG ACACCA CGATGC CTGTAG CAATGG CAACAA CGTTGC

1141 GCAAAC TATTAA CTGGCG AACTAC TTACTC TAGCTT CCCGGC AACAAT TAATAG ACTGGA

1201 TGGAGG CGGATA AAGTTG CAGGAC CACTTC TGCGCT CGGCCC TTCCGG CTGGCT GGTTTA

1261 TTGCTG ATAAAT CTGGAG CCGGTG AGCGTG GGTCTC GCGGTA TCATTG CAGCAC TGGGGC

1321 CAGATG GTAAGC CCTCCC GTATCG TAGTTA TCTACA CGACGG GGAGTC AGGCAA CTATGG

1381 ATGAAC GAAATA GACAGA TCGCTG AGATAG GTGCCT CACTGA TTAAGC ATTGGT AACTGT

1441 CAGACC AAGTTT ACTCAT ATATAC TTTAGA TTGATT TAAAAC TTCATT TTTAAT TTAAAA

1501 GGATCT AGGTGA AGATCC TTTTTG ATAATC TCATGA CCAAAA TCCCTT AACGTG AGTTTT

1561 CGTTCC ACTGAG CGTCAG ACCCCG TAGAAA AGATCA AAGGAT CTTCTT GAGATC CTTTTT

1621 TTCTGC GCGTAA TCTGCT GCTTGC AAACAA AAAAAC CACCGC TACCAG CGGTGG TTTGTT

1681 TGCCGG ATCAAG AGCTAC CAACTC TTTTTC CGAAGG TAACTG GCTTCA GCAGAG CGCAGA

1741 TACCAA ATACTG TTCTTC TAGTGT AGCCGT AGTTAG GCCACC ACTTCA AGAACT CTGTAG

1801 CACCGC CTACAT ACCTCG CTCTGC TAATCC TGTTAC CAGTGG CTGCTG CCAGTG GCGATA

1861 AGTCGT GTCTTA CCGGGT TGGACT CAAGAC GATAGT TACCGG ATAAGG CGCAGC GGTCGG

1921 GCTGAA CGGGGG GTTCGT GCACAC AGCCCA GCTTGG AGCGAA CGACCT ACACCG AACTGA

1981 GATACC TACAGC GTGAGC TATGAG AAAGCG CCACGC TTCCCG AAGGGA GAAAGG CGGACA

2041 GGTATC CGGTAA GCGGCA GGGTCG GAACAG GAGAGC GCACGA GGGAGC TTCCAG GGGGAA

2101 ACGCCT GGTATC TTTATA GTCCTG TCGGGT TTCGCC ACCTCT GACTTG AGCGTC GATTTT

2161 TGTGAT GCTCGT CAGGGG GGCGGA GCCTAT GGAAAA ACGCCA GCAACG CGGCCT TTTTAC

2221 GGTTCC TGGCCT TTTGCT GGCCTT TTGCTC ACATGT TCTTTC CTGCGT TATCCC CTGATT

2281 CTGTGG ATAACC GTATTA CCGCCT TTGAGT GAGCTG ATACCG CTCGCC GCAGCC GAACGA

2341 CCGAGC GCAGCG AGTCAG TGAGCG AGGAAG CGGAAG AGCGCC CAATAC GCAAAC CGCCTC

2401 TCCCCG CGCGTT GGCCGA TTCATT AATGCA GCTGGC ACGACA GGTTTC CCGACT GGAAAG

2461 CGGGCA GTGAGC GCAACG CAATTA ATGTGA GTTAGC TCACTC ATTAGG CACCCC AGGCTT

2521 TACACT TTATGC TTCCGG CTCGTA TGTTGT GTGGAA TTGTGA GCGGAT AACAAT TTCACA

2581 CAGGAA ACAGCT ATGACC ATGATT ACGCCA AGCTCG AAATTA ACCCTC ACTAAA GGGAAC

2641 AAAAGC TGGTAC GCGGCC GCTCGT CGTCTG ATTGGC TCTCGG GGCCCA GAAAAC TGGCCC

2701 TTGCCA TTGGCT CGTGTT CGTGCA AGTTGA GTCCAT CCGCCG GCCAGC GGGGGC GGCGAG

2761 GAGGCG CTCCCA GGTTCC GGCCCT CCCCTC GGCCCC GCGCCG CAGAGT CTGGCC GCGCGC

2821 CCCTGC GCAACG TGGCAG GAAGCG CGCGCT GGGGGC GGGGAC GGGCAG TAGGGC TGAGCG

2881 GCTGCG GGGCGG GTGCAA GCACGT TTCCGA CTTGAG TTGCCT CAAGAG GGGCGT GCTGAG

2941 CCAGAC CTCCAT CGCGCA CTCCGG GGAGTG GAGGGA AGGAGC GAGGGC TCAGTT GGGCTG

3001 TTTTGG AGGCAG GAAGCA CTTGCT CTCCCA AAGTCG CTCTGA GTTGTT ATCAGT AAGGGA

3061 GCTGCA GTGGAG TAGGCG GGGAGA AGGCCG CACCCT TCTCCG GAGGGG GGAGGG GAGTGT

3121 TGCAAT ACCTTT CTGGGA GTTCTC TGCTGC CTCCTG GCTTCT GAGGAC CGCCCT GGGCCT

3181 GGGAGA ATCCCT TCCCCC TCTTCC CTCGTG ATCTGC AACTCC AGTCTT TCTAGA AGATGG

3241 GCGGGA GTCTTC TGGGCA GGCTTA AAGGCT AACCTG GTGTGT GGGCGT TGTCCT GCAGGG

3301 GAATTG AACAGG TGTAAA ATTGGA GGGACA AGACTT CCCACA GATTTT CGGTTT TGTCGG

3361 GAAGTT TTTTAA TAGGGG CAAATA AGGAAA ATGGGA GGATAG GTAGTC ATCTGG GGTTTT

3421 ATGCAG CAAAAC TACAGG TTATTA TTGCTT GTGATC CGCCTC GGAGTA TTTTCC ATCGAG

3481 GTAGAT TAAAGA CATGCT CACCCG AGTTTT ATACTC TCCTGC TTGAGA TCCTTA CTACAG

3541 TATGAA ATTACA GTGTCG CGAGTT AGACTA TGTAAG CAGAAT TTTAAT CATTTT TAAAGA

3601 GCCCAG TACTTC ATATCC ATTTCT CCCGCT CCTTCT GCAGCC TTATCA AAAGGT ATTTTA

3661 GAACAC TCATTT TAGCCC CATTTT CATTTA TTATAC TGGCTT ATCCAA CCCCTA GACAGA

3721 GCATTG GCATTT TCCCTT TCCTGA TCTTAG AAGTCT GATGAC TCATGA AACCAG ACAGAT

3781 TAGTTA CATACA CCACAA ATCGAG GCTGTA GCTGGG GCCTCA ACACTG CAGTTC TTTTAT

3841 AACTCC TTAGTA CACTTT TTGTTG ATCCTT TGCCTT GATCCT TAATTT TCAGTG TCTATC

3901 ACCTCT CCCGTC AGGTGG TGTTCC ACATTT GGGCCT ATTCTC AGTCCA GGGAGT TTTACA

3961 ACAATA GATGTA TTGAGA ATCCAA CCTAAA GCTTAA CTTTCC ACTCCC ATGAAT GCCTCT

4021 CTCCTT TTTCTC CATTTA TAAACT GAGCTA TTAACC ATTAAT GGTTTC CAGGTG GATGTC

4081 TCCTCC CCCAAT ATTACC TGATGT ATCTTA CATATT GCCAGG CTGATA TTTTAA GACATT

4141 AAAAGG TATATT TCATTA TTGAGC CACATG GTATTG ATTACT GCTTAC TAAAAT TTTGTC

4201 ATTGTA CACATC TGTAAA AGGTGG TTCCTT TTGGAA TGCAAA GTTCAG GTGTTT GTTGTC

4261 TTTCCT GACCTA AGGTCT TGTGAG CTTGTA TTTTTT CTATTT AAGCAG TGCTTT CTCTTG

4321 GACTGG CTTGAC TCATGG CATTCT ACACGT TATTGC TGGTCT AAATGT GATTTT GCCAAG

4381 CTTCTT CAGGAC CTATAA TTTTGC TTGACT TGTAGC CAAACA CAAGTA AAATGA TTAAGC

4441 AACAAA TGTATT TGTGAA GCTTGG TTTTTA GGTTGT TGTGTT GTGTGT GCTTGT GCTCTA

4501 TAATAA TACTAT CCAGGG GCTGGA GAGGTG GCTCGG AGTTCA AGAGCA CAGACT GCTCTT

4561 CCAGAA GTCCTG AGTTCA ATTCCC AGCAAC CACATG GTGGCT CACAAC CATCTG TAATGG

4621 GATCTG ATGCCC TCTTCT GGTGTG TCTGAA GACCAC AAGTGT ATTCAC ATTAAA TAAATA

4681 AATCCT CCTTCT TCTTCT TTTTTT TTTTTT TAAAGA GAATAC TGTCTC CAGTAG AATTTA

4741 CTGAAG TAATGA AATACT TTGTGT TTGTTC CAATAT GGTAGC CAATAA TCAAAT TACTCT

4801 TTAAGC ACTGGA AATGTT ACCAAG GAACTA ATTTTT ATTTGA AGTGTA ACTGTG GACAGA

4861 GGAGCC ATAACT GCAGAC TTGTGG GATACA GAAGAC CAATGC AGACTT TAATGT CTTTTC

4921 TCTTAC ACTAAG CAATAA AGAAAT AAAAAT TGAACT TCTAGT ATCCTA TTTGTT TAAACT

4981 GCTAGC TTTACT TAACTT TTGTGC TTCATC TATACA AAGCTG AAAGCT AAGTCT GCAGCC

5041 ATTACT AAACAT GAAAGC AAGTAA TGATAA TTTTGG ATTTCA AAAATG TAGGGC CAGAGT

5101 TTAGCC AGCCAG TGGTGG TGCTTG CCTTTA TGCCTT TAATCC CAGCAC TCTGGA GGCAGA

5161 GACAGG CAGATC TCTGAG TTTGAG CCCAGC CTGGTC TACACA TCAAGT TCTATC TAGGAT

5221 AGCCAG GAATAC ACACAG AAACCC TGTTGG GGAGGG GGGCTC TGAGAT TTCATA AAATTA

5281 TAATTG AAGCAT TCCCTA ATGAGC CACGTC GACATT GATTAT TGACTA GTTATT AATAGT

5341 AATCAA TTACGG GGTCAT TAGTTC ATAGCC CATATA TGGAGT TCCGCG TTACAT AACTTA

5401 CGGTAA ATGGCC CGCCTG GCTGAC CGCCCA ACGACC CCCGCC CATTGA CGTCAA TAATGA

5461 CGTATG TTCCCA TAGTAA CGCCAA TAGGGA CTTTCC ATTGAC GTCAAT GGGTGG AGTATT

5521 TACGGT AAACTG CCCACT TGGCAG TACATC AAGTGT ATCATA TGCCAA GTACGC CCCCTA

5581 TTGACG TCAATG ACGGTA AATGGC CCGCCT GGCATT ATGCCC AGTACA TGACCT TATGGG

5641 ACTTTC CTACTT GGCAGT ACATCT ACGTAT TAGTCA TCGCTA TTACCA TGGTCG AGGTGA

5701 GCCCCA CGTTCT GCTTCA CTCTCC CCATCT CCCCCC CCTCCC CACCCC CAATTT TGTATT

5761 TATTTA TTTTTT AATTAT TTTGTG CAGCGA TGGGGG CGGGGG GGGGGG GGGGGC GCGCGC

5821 CAGGCG GGGCGG GGCGGG GCGAGG GGCGGG GCGGGG CGAGGC GGAGAG GTGCGG CGGCAG

5881 CCAATC AGAGCG GCGCGC TCCGAA AGTTTC CTTTTA TGGCGA GGCGGC GGCGGC GGCGGC

5941 CCTATA AAAAGC GAAGCG CGCGGC GGGCGG GAGTCG CTGCGC GCTGCC TTCGCC CCGTGC

6001 CCCGCT CCGCCG CCGCCT CGCGCC GCCCGC CCCGGC TCTGAC TGACCG CGTTAC TCCCAC

6061 AGGTGA GCGGGC GGGACG GCCCTT CTCCTC CGGGCT GTAATT AGCGCT TGGTTT AATGAC

6121 GGCTTG TTTCTT TTCTGT GGCTGC GTGAAA GCCTTG AGGGGC TCCGGG AGGGCC CTTTGT

6181 GCGGGG GGAGCG GCTCGG GGGGTG CGTGCG TGTGTG TGTGCG TGGGGA GCGCCG CGTGCG

6241 GCTCCG CGCTGC CCGGCG GCTGTG AGCGCT GCGGGC GCGGCG CGGGGC TTTGTG CGCTCC

6301 GCAGTG TGCGCG AGGGGA GCGCGG CCGGGG GCGGTG CCCCGC GGTGCG GGGGGG GCTGCG

6361 AGGGGA ACAAAG GCTGCG TGCGGG GTGTGT GCGTGG GGGGGT GAGCAG GGGGTG TGGGCG

6421 CGTCGG TCGGGC TGCAAC CCCCCC TGCACC CCCCTC CCCGAG TTGCTG AGCACG GCCCGG

6481 CTTCGG GTGCGG GGCTCC GTACGG GGCGTG GCGCGG GGCTCG CCGTGC CGGGCG GGGGGT

6541 GGCGGC AGGTGG GGGTGC CGGGCG GGGCGG GGCCGC CTCGGG CCGGGG AGGGCT CGGGGG

6601 AGGGGC GCGGCG GCCCCC GGAGCG CCGGCG GCTGTC GAGGCG CGGCGA GCCGCA GCCATT

6661 GCCTTT TATGGT AATCGT GCGAGA GGGCGC AGGGAC TTCCTT TGTCCC AAATCT GTGCGG

6721 AGCCGA AATCTG GGAGGC GCCGCC GCACCC CCTCTA GCGGGC GCGGGG CGAAGC GGTGCG

6781 GCGCCG GCAGGA AGGAAA TGGGCG GGGAGG GCCTTC GTGCGT CGCCGC GCCGCC GTCCCC

6841 TTCTCC CTCTCC AGCCTC GGGGCT GTCCGC GGGGGG ACGGCT GCCTTC GGGGGG GACGGG

6901 GCAGGG CGGGGT TCGGCT TCTGGC GTGTGA CCGGCG GCTCTA GAGCCT CTGCTA ACCATG

6961 TTCATG CCTTCT TCTTTT TCCTAC AGCTCC TGGGCA ACGTGC TGGTTA TTGTGC TGTCTC

7021 ATCATT TTGGCA AAGAAT TGCATC AGCGCT AGCAAT TCCTGT GGCATT GCTGCA ATTCGG

7081 CTCCGC GAACCT TACTCT CCGAAG ACTTAA CTCGAC CGTTCA TCCACG AAGCTT ACGCCA

7141 GAATTG CGAGGT ATCGCA TTATCG ATCGAT AAGCTA GCTTGG GCTGCA GGTCGA GGGACC

7201 TAATAA CTTCGT ATAGCA TACATT ATACGA AGTTAT ATTAAG GGTTCC GGATCA GCTTGA

7261 TGGGGA TCCAGA CATGAT AAGATA CATTGA TGAGTT TGGACA AACCAC AACTAG AATGCA

7321 GTGAAA AAAATG CTTTAT TTGTGA AATTTG TGATGC TATTGC TTTATT TGTAAC CATTAT

7381 AAGCTG CAATAA ACAAGT TAACAA CAACAA TTGCAT TCATTT TATGTT TCAGGT TCAGGG

7441 GGAGGT GTGGGA GGTTTT TTAAAG CAAGTA AAACCT CTACAA ATGTGG TATGGC TGATTA

7501 TGATCC TCTAGA GTCGCA GATCCA GACATG ATAAGA TACATT GATGAG TTTGGA CAAACC

7561 ACAACT AGAATG CAGTGA AAAAAA TGCTTT ATTTGT GAAATT TGTGAT GCTATT GCTTTA

7621 TTTGTA ACCATT ATAAGC TGCAAT AAACAA GTTAAC AACAAC AATTGC ATTCAT TTTATG

7681 TTTCAG GTTCAG GGGGAG GTGTGG GAGGTT TTTTAA AGCAAG TAAAAC CTCTAC AAATGT

7741 GGTATG GCTGAT TATGAT CCTCTA GAGTCG CAGATC CAGACA TGATAA GATACA TTGATG

7801 AGTTTG GACAAA CCACAA CTAGAA TGCAGT GAAAAA AATGCT TTATTT GTGAAA TTTGTG

7861 ATGCTA TTGCTT TATTTG TAACCA TTATAA GCTGCA ATAAAC AAGTTA ACAACA ACAATT

7921 GCATTC ATTTTA TGTTTC AGGTTC AGGGGG AGGTGT GGGAGG TTTTTT AAAGCA AGTAAA

7981 ACCTCT ACAAAT GTGGTA TGGCTG ATTATG ATCCTC TAGAGT CGCAGA TCCTCT AGAGTC

8041 GCAGAT CTGCAA GCTAAT TCCTGC AGGTCG AGGGAC CTAATA ACTTCG TATAGC ATACAT

8101 TATACG AAGTTA TATTAA GGGTTC CGGATC CACTAC ACGTGG CCACCA TGGCCG CCGCCG

8161 GCGCCC GGCTCA GCCCCG GCCCCG GCTCGG GGCTCC GGGGGC GGCCGA GGCTCT GCTTCC

8221 ACCCGG GGCCGC CGCCAC TGCTGC CGCTGC TGCTGC TGTTCC TGCTCC TGCTGC CGCCGC

8281 CGCCGC TGCTGG CCGGCG CCACCG CCGCTG CCTCGC GGGAGC CCGACA GCCCGT GCCGGC

8341 TGAAGA CCGTCA CGGTGT CCACAC TGCCCG CCCTGC GGGAGA GCGACA TCGGCT GGAGCG

8401 GCGCCC GCGCCG GGGCCG GGGCTG GGACCG GGGCCG GAGCCG CCGCCG CCGCCG CGTCCC

8461 CGGGCT CTCCTG GCTCTG CCGGCA CCGCCG CCGAGT CGCGCC TCCTGC TCTTTG TGCGTA

8521 ACGAGC TGCCGG GGCGCA TCGCGG TGCAGG ACGACC TGGACA ACACCG AGCTGC CCTTCT

8581 TCACCC TGGAGA TGTCTG GCACAG CGGCGG ACATCT CGCTGG TGCACT GGAGAC AGCAGT

8641 GGCTGG AGAATG GCACCT TGTACT TCCACG TCTCCA TGAGCA GCTCCG GGCAGC TGGCCC

8701 AAGCCA CCGCCC CCACTC TCCAGG AGCCCT CGGAGA TTGTTG AGGAGC AGATGC ACATCC

8761 TCCACA TTTCTG TGATGG GTGGCC TCATCG CGCTGC TGCTGC TGCTGC TGGTGT TCACCG

8821 TGGCGC TGTACG CCCAGC GACGTT GGCAGA AGCGTC GCCGCA TCCCCC AGAAGA GCGCAA

8881 GCACAG AAGCCA CTCATG AGATCC ACTACA TCCCAT CTGTGC TGCTGG GTCCCC AGGCGC

8941 GGGAGA GCTTCC GTTCAT CCCGGC TGCAAA CCCACA ATTCCG TCATTG GCGTGC CCATCC

9001 GGGAGA CTCCCA TCCTGG ATGACT ATGACT GTGAGG AGGATG AGGAGC CACCTA GGCGGG

9061 CCAACC ATGTCT CCCGCG AGGACG AGTTTG GCAGCC AGGTGA CCCACA CTCTGG ACAGTC

9121 TGGGAC ATCCAG GGGAAG AGAAGG TGGACT TTGAGA AGAAAG CAGCAG CTGAGG CGACTC

9181 AGGAGA CAGTGG AGTCCC TGATGC AGAAGT TCAAGG AGAGTT TCCGCG CTAACA CGCCCA

9241 TCGAGA TCGGTC AGCTGC AACCAC CCCTGC GCAGCA CATCGG CAGGGA AGAGGA AGCGGA

9301 GGAGCA AGTCTC GAGGAG GAATCA GCTTTG GGAGAG CCAAGG GGACGT CGGGCT CAGAGG

9361 CAGACG ATGAAA CTCAGC TGACAT TCTACA CGGAGC AGTACC GCAGTC GCCGCC GCAGCA

9421 AAGGTT TGCTGA AAAGCC CAGTGA ACAAGA CAGCCC TGACAC TGATTG CTGTGA GTTCCT

9481 GCATCC TGGCCA TGGTGT GTGGCA GCCAGA TGTCTT GTCCAC TCACTG TGAAGG TGACTC

9541 TGCATG TGCCCG AGCACT TCATAG CAGATG GAAGCA GCTTCG TGGTGA GTGAAG GGAGCT

9601 ACCTGG ACATCT CCGACT GGTTAA ACCCAG CCAAGC TTTCCC TGTATT ACCAGA TCAATG

9661 CCACCT CCCCAT GGGTGA GGGACC TCTGTG GACAAA GGACGA CAGATG CCTGTG AGCAGC

9721 TCTGCG ACCCAG AAACCG GAGAGT GCAGCT GTCATG AAGGCT ATGCCC CTGACC CTGTTC

9781 ACAGAC ACCTGT GTGTGC GCAGTG ACTGGG GACAGA GTGAAG GACCTT GGCCCT ACACGA

9841 CACTTG AGAGGG GCTATG ATCTGG TGACAG GGGAGC AAGCCC CTGAAA AGATTC TCAGGT

9901 CTACTT TCAGCT TGGGCC AAGGCC TCTGGC TTCCTG TCAGCA AAAGCT TTGTGG TTCCGC

9961 CTGTGG AGCTGT CCATCA ACCCCC TGGCCA GCTGCA AGACCG ATGTGC TCGTCA CGGAAG

10021 ACCCTG CAGATG TCAGGG AAGAAG CGATGC TGTCCA CATACT TTGAAA CCATCA ATGACC

10081 TGCTGT CTTCCT TCGGGC CAGTTC GTGACT GCTCTC GGAACA ATGGGG GCTGCA CTCGCA

10141 ACTTCA AGTGTG TGTCTG ACCGGC AGGTGG ATTCCT CGGGAT GTGTGT GCCCTG AGGAGC

10201 TGAAAC CCATGA AGGATG GCTCTG GCTGCT ACGACC ACTCCA AAGGCA TTGACT GCTCTG

10261 ATGGCT TTAATG GCGGCT GTGAGC AGCTGT GCCTGC AGCAGA CGCTGC CCCTGC CCTACG

10321 ATGCCA CTTCGA GCACCA TCTTCA TGTTCT GCGGTT GCGTGG AGGAGT ACAAAC TGGCTC

10381 CTGATG GAAAAT CCTGCT TAATGC TCTCAG ATGTCT GCGAGG GCCCCA AGTGCC TCAAAC

10441 CTGACT CCAAAT TCAATG ATACCC TCTTTG GAGAGA TGCTAC ATGGTT ACAACA ACCGGA

10501 CCCAGC ATGTGA ACCAAG GCCAAG TCTTCC AGATGA CCTTTA GGGAGA ACAACT TCATCA

10561 AGGACT TTCCCC AGCTGG CCGATG GGCTGT TGGTGA TCCCGC TGCCGG TGGAGG AGCAGT

10621 GCCGGG GGGTCC TCTCCG AGCCCC TTCCGG ACCTCC AACTGC TCACTG GAGATA TCAGGT

10681 ATGATG AGGCCA TGGGTT ACCCCA TGGTGC AGCAGT GGCGGG TCCGGA GCAACC TCTACC

10741 GTGTGA AGCTCA GCACCA TCACCC TCGCAG CAGGCT TCACTA ATGTTC TCAAGA TTCTGA

10801 CCAAGG AGAGCA GTCGGG AGGAGC TGCTGT CCTTCA TCCAGC ACTATG GCTCCC ACTACA

10861 TCGCAG AGGCCC TCTATG GCTCAG AGCTCA CCTGCA TCATCC ACTTTC CCAGCA AGAAGG

10921 TCCAGC AGCAGC TGTGGC TCCAGT ATCAGA AAGAGA CCACAG AGCTGG GCAGCA AGAAGG

10981 AGCTCA AGTCCA TGCCCT TCATCA CCTACC TCTCAG GTTTGC TGACAG CCCAGA TGCTGT

11041 CAGATG ACCAGC TCATTT CAGGTG TGGAGA TTCGCT GTGAGG AGAAGG GGCGCT GTCCAT

11101 CTACCT GTCACC TTTGCC GCCGGC CAGGCA AGGAGC AGCTGA GCCCCA CACCAG TGCTGC

11161 TGGAAA TCAACC GTGTGG TGCCAC TTTATA CCCTCA TCCAAG ACAATG GCACAA AGGAGG

11221 CCTTCA AGAGTG CACTGA TGAGTT CCTACT GGTGCT CAGGGA AAGGGG ATGTGA TCGATG

11281 ACTGGT GCAGGT GTGACC TCAGCG CCTTTG ATGCCA ATGGGC TCCCCA ACTGCA GCCCCC

11341 TTCTGC AGCCGG TGCTGC GGCTGT CCCCAA CAGTGG AGCCCT CCAGTA CTGTGG TCTCCT

11401 TGGAGT GGGTGG ATGTTC AGCCAG CTATTG GGACCA AGGTCT CCGACT ATATTC TGCAGC

11461 ATAAGA AAGTGG ATGAAT ACACAG ACACTG ACCTGT ACACAG GAGAAT TCCTGA GTTTTG

11521 CTGATG ACTTAC TCTCTG GCCTGG GCACAT CTTGTG TAGCAG CTGGTC GAAGCC ATGGAG

11581 AGGTCC CTGAAG TCAGTA TCTACT CAGTCA TCTTCA AGTGTC TGGAGC CCGACG GTCTCT

11641 ACAAGT TCACTC TGTATG CTGTGG ATACAC GAGGGA GGCACT CAGAGC TAAGCA CGGTGA

11701 CCCTGA GGACGG CCTGTC CACTGG TAGATG ACAACA AGGCAG AAGAAA TAGCTG ACAAGA

11761 TCTACA ATCTGT ACAATG GGTACA CAAGTG GAAAGG AGCAGC AGATGG CCTACA ACACAC

11821 TGATGG AGGTCT CAGCCT CGATGC TGTTCC GAGTCC AGCACC ACTACA ACTCTC ACTATG

11881 AAAAGT TTGGCG ACTTCG TCTGGA GAAGTG AGGATG AGCTGG GGCCCA GGAAGG CCCACC

11941 TGATTC TACGGC GACTGG AGAGGG TGAGTA GCCACT GCTCCA GCCTCC TGCGGA GTGCCT

12001 ACATCC AGAGCC GCGTGG AAACAG TGCCCT ATCTTT TCTGCC GCAGCG AGGAGG TCCGGC

12061 CTGCAG GCATGG TGTGGT ATAGCA TCCTCA AGGACA CCAAAA TCACGT GTGAGG AGAAGA

12121 TGGTGT CAATGG CCCGAA ACACGT ACGGGG AGTCCA AGGGCC GGAGCC AGTGTC CTGGGC

12181 TAAACA CAAGAG TGCTGA TTCCCA CTGTAA GTTACA GTGAAG AACTTC TGCTAT CTGAGG

12241 GCATGT GTTTTC ATCTTC AAAAAA GGATGG ACAGTC CCCATG AACCTT CCCTCT CCAACC

12301 ACACAG GCCTTG CTTCTG GACATG CAGTGA TAACTC TCTGTT TGCTGG ATGAAG ATCATG

12361 TTGGCT CTATGC ACATTC AGATAA CCTTCT ACACCA GACACC CCTGGT GAGCGA TCGCAC

12421 CCGCGG ACTAGA GCTCGC TGATCA GCCTCG ACTGTG CCTTCT AGTTGC CAGCCA TCTGTT

12481 GTTTGC CCCTCC CCCGTG CCTTCC TTGACC CTGGAA GGTGCC ACTCCC ACTGTC CTTTCC

12541 TAATAA AATGAG GAAATT GCATCG CATTGT CTGAGT AGGTGT CATTCT ATTCTG GGGGGT

12601 GGGGTG GGGCAG GACAGC AAGGGG GAGGAT TGGGAA GACAAT AGCAGG CATGCT GGGGAT

12661 GCGGTG GGCTCT ATGGCT TCTGAG GCGGAA AGAACC AGCTGG GGGATA TCATTC TGGTGT

12721 CGACGC TACTAA CTTTAA ATAATT GGCATT ATTTAA AGTTAA TCAAGC TTAGCT TGATAT

12781 CGAATT CCGAAG TTCCTA TTCTCT AGAAAG TATAGG AACTTC AGGTCT GAAGAG GAGTTT

12841 ACGTCC AGCCAA GCTAGC TCCATG GGCCAG GCAAAT ATCCCT TACCAG CCTCAC AGAGAC

12901 CTCCCC CACCCC CCGCAA CCCTAG AGTTCT TTTACT AGTGAG GGACAA GTGGAC AATGGT

12961 GCTGTT GTGGGC CCCACC CTGTGT CCCCTG TGCCCA CAGTGG TCACTC TGCTTG GCAGGC

13021 AGGTGT TGCAGG CTGGCT GCTCCA GGCCCT GGCAGG AGGTAC TGAAGG ACCTGG TAGGCT

13081 CAGATG CCCTGG ATGCCA AGGCAC TGCTGG AGTACT TCCAAC CGGTCA GCCAGT GGCTGG

13141 AAGAGC AGAATC AGCGGA ATGGCG AAGTCC TAGGCT GGCCAG AGAATC AGTGGC GTCCAC

13201 CGTTAC CCGACA ACTATC CAGAGG GCATTG GTAAAG CTCTGA GTGAGG GTGGAC TGGGAC

13261 CAAGAG AAGTCC TGGCCT CTGGCC TCTGGC TTCTGG GTCAAA GCCTCA GCATCC TGGTCA

13321 CTTTGC TGCCAG CTGAGC CCCAGT GTCCTT TGCTTC AGTGCC AAGCCA CCCCTG GGCTCA

13381 TCCTCA GGGCCC TAAGCA GAAATG GGTATG TCTTTC TCTCAG GGTCCT AGAGAC AGTGTG

13441 CCCAAG CCTGAG GGCCCT TGGGGT CAGGCT GGCTGG CACATT GCTCTA TGAGGT CACACT

13501 GCAGGC TTGGCT CTTATT GGCCGG TGATGG GAGCTT CAGGGC TCTGCT TTCCTG CGGCCA

13561 TGCCTA AGAAGA AGAGGA AGGTTT CTGAGC TGATTA TTAGTG GTTCAT CTGGTG GATTCC

13621 TGCGAA ACATCG GCAAAG AGTATC AGGAGG CCGCTG AAAACT TCATGA GGTTTA TGAATG

13681 ACCAGG GGGCGT ACGCTC CTAACA CTTTGA GGGATT TGAGGT TGGTCT TTCATA GCTGGG

13741 CCAGAT GGTGCC ATGCTC GGCAGC TTGCAT GGTTTC CAATTA GTCCTG AAATGG CACGCG

13801 AATACT TTCTTC AGTTGC ACGATG CAGACC TGGCCT CCACTA CCATCG ACAAGC ACTATG

13861 CTATGC TTAATA TGCTTC TGTCCC ACTGCG GACTGC CACCCT TGTCCG ACGACA AGTCAG

13921 TGAGTC TTGCCA TGAGAA GAATTA GAAGAG AAGCCG CAACCG AAAAGG GTGAGA GGACAG

13981 GACAGG CAATCC CCCTGC GCTGGG ACGACC TGAAGC TGCTGG ATGTGC TGCTCA GCAGGA

14041 GCGAGC GGCTGG TCGACC TGCGCA ACAGGG CTTTCC TGTTCG TAGCCT ATAACA CCCTCA

14101 TGAGAA TGTCTG AAATAT CACGCA TCAGGG TTGGGG ACTTGG ATCAGA CAGGAG ACACAG

14161 TGACCC TGCACA TCAGTC ACACTA AGACAA TCACCA CAGCTG CGGGCC TTGACA AAGTGC

14221 TCTCCC GGCGAA CCACAG CAGTGC TCAATG ACTGGC TGGACG TCAGTG GGCTTA GAGAAC

14281 ATCCAG ACGCTG TGCTCT TCCCAC CTATAC ACCGGT CAAACA AAGCCC GCATTA CTACCA

14341 CGCCCC TGACCG CCCCTG CCATGG AGAAGA TTTTCA GTGATG CCTGGG TGCTGC TGAACA

14401 AACGGG ACGCCA CCCCCA ATAAAG GGAGGT ATAGGA CCTGGA CCGGCC ATTCCG CCAGGG

14461 TGGGTG CCGCAA TAGACA TGGCCG AGAAAC AGGTGT CTATGG TCGAGA TTATGC AGGAAG

14521 GGACAT GGAAGA AGCCTG AAACAC TGATGC GGTATC TCAGAA GGGGCG GAGTGT CCGTGG

14581 GAGCCA ATTCTC GACTGA TGGATA GCTAAA CTTGTT TATTGC AGCTTA TAATGG TTACAA

14641 ATAAAG CAATAG CATCAC AAATTT CACAAA TAAAGC ATTTTT TTCACT GCATTC TAGTTG

14701 TGGTTT GTCCAA ACTCAT CAATGT ATCTTA TCATGT CTGGCT CTAGCG CTTTGG CTGCAG

14761 GTCGTC GAAATT CTACCG GGTAGG GGAGGC GCTTTT CCCAAG GCAGTC TGGAGC ATGCGC

14821 TTTAGC AGCCCC GCTGGG CACTTG GCGCTA CACAAG TGGCCT CTGGCC TCGCAC ACATTC

14881 CACATC CACCGG TAGGCG CCAACC GGCTCC GTTCTT TGGTGG CCCCTT CGCGCC ACCTTC

14941 TACTCC TCCCCT AGTCAG GAAGTT CCCCCC CGCCCC GCAGCT CGCGTC GTGCAG GACGTG

15001 ACAAAT GGAAGT AGCACG TCTCAC TAGTCT CGTGCA GATGGA CAGCAC CGCTGA GCAATG

15061 GAAGCG GGTAGG CCTTTG GGGCAG CGGCCA ATAGCA GCTTTG CTCCTT CGCTTT CTGGGC

15121 TCAGAG GCTGGG AAGGGG TGGGTC CGGGGG CGGGCT CAGGGG CGGGCT CAGGGG CGGGGC

15181 GGGCGC CCGAAG GTCCTC CGGAGG CCCGGC ATTCTG CACGCT TCAAAA GCGCAC GTCTGC

15241 CGCGCT GTTCTC CTCTTC CTCATC TCCGGG CCTTTC GACCTG CAGCCT GTTGAC AATTAA

15301 TCATCG GCATAG TATATC GGCATA GTATAA TACGAC AAGGTG AGGAAC TAAACC ATGGGA

15361 TCGGCC ATTGAA CAAGAT GGATTG CACGCA GGTTCT CCGGCC GCTTGG GTGGAG AGGCTA

15421 TTCGGC TATGAC TGGGCA CAACAG ACAATC GGCTGC TCTGAT GCCGCC GTGTTC CGGCTG

15481 TCAGCG CAGGGG CGCCCG GTTCTT TTTGTC AAGACC GACCTG TCCGGT GCCCTG AATGAA

15541 CTGCAG GACGAG GCAGCG CGGCTA TCGTGG CTGGCC ACGACG GGCGTT CCTTGC GCAGCT

15601 GTGCTC GACGTT GTCACT GAAGCG GGAAGG GACTGG CTGCTA TTGGGC GAAGTG CCGGGG

15661 CAGGAT CTCCTG TCATCT CACCTT GCTCCT GCCGAG AAAGTA TCCATC ATGGCT GATGCA
15721 ATGCGG CGGCTG CATACG CTTGAT CCGGCT ACCTGC CCATTC GACCAC CAAGCG AAACAT
15781 CGCATC GAGCGA GCACGT ACTCGG ATGGAA GCCGGT CTTGTC GATCAG GATGAT CTGGAC
15841 GAAGAG CATCAG GGGCTC GCGCCA GCCGAA CTGTTC GCCAGG CTCAAG GCGCGC ATGCCC
15901 GACGGC GATGAT CTCGTC GTGACC CATGGC GATGCC TGCTTG CCGAAT ATCATG GTGGAA
15961 AATGGC CGCTTT TCTGGA TTCATC GACTGT GGCCGG CTGGGT GTGGCG GACCGC TATCAG
16021 GACATA GCGTTG GCTACC CGTGAT ATTGCT GAAGAG CTTGGC GGCGAA TGGGCT GACCGC
16081 TTCCTC GTGCTT TACGGT ATCGCC GCTCCC GATTCG CAGCGC ATCGCC TTCTAT CGCCTT
16141 CTTGAC GAGTTC TTCTGA GGGGAT CAATTC TCTAGA GCTCGC TGATCA GCCTCG ACTGTG
16201 CCTTCT AGTTGC CAGCCA TCTGTT GTTTGC CCCTCC CCCGTG CCTTCC TTGACC CTGGAA
16261 GGTGCC ACTCCC ACTGTC CTTTCC TAATAA AATGAG GAAATT GCATCG CATTGT CTGAGT
16321 AGGTGT CATTCT ATTCTG GGGGGT GGGGTG GGGCAG GACAGC AAGGGG GAGGAT TGGGAA
16381 GACAAT AGCAGG CATGCT GGGGAT GCGGTG GGCTCT ATGGCT TCTGAG GCGGAA AGAACC
16441 AGCTGG GGCTCG ACTAGA GCTTGC GGAACC CTTCGA AGTTCC TATTCT CTAGAA AGTATA
16501 GGAACT TCATCA GTCAGG TACATA ACTAAC TTTAAA TAATTG GCATTA TTTAAA GTTACT
16561 CGAGTA TGGATG TGGCTA AATCCG TCTACC TTTCTG ATGAGA TTTGGG TATTAT TTTTTC
16621 TGTCTC TGCTGT TGGTTG GGTCTT TTGACA CTGTGG GCTTTC TTTAAA GCCTCC TTCCTG
16681 CCATGT GGTCTC TTGTTT GCTACT AACTTC CCATGG CTTAAA TGGCAT GGCTTT TTGCCT
16741 TCTAAG GGCAGC TGCTGA GATTTG CAGCCT GATTTC CAGGGT GGGGTT GGGAAA TCTTTC
16801 AAACAC TAAAAT TGTCCT TTAATT TTTTTT TTAAAA AATGGG TTATAT AATAAA CCTCAT
16861 AAAATA GTTATG AGGAGT GAGGTG GACTAA TATTAA ATGAGT CCCTCC CCTATA AAAGAG
16921 CTATTA AGGCTT TTTGTC TTATAC TTAACT TTTTTT TTAAAT GTGGTA TCTTTA GAACCA
16981 AGGGTC TTAGAG TTTTAG TATACA GAAACT GTTGCA TCGCTT AATCAG ATTTTC TAGTTT
17041 CAAATC CAGAGA ATCCAA ATTCTT CACAGC CAAAGT CAAATT AAGAAT TTCTGA CTTTTA
17101 ATGTTA ATTTGC TTACTG TGAATA TAAAAA TGATAG CTTTTC CTGAGG CAGGGT CTCACT
17161 ATGTAT CTCTGC CTGATC TGCAAC AAGATA TGTAGA CTAAAG TTCTGC CTGCTT TTGTCT
17221 CCTGAA TACTAA GGTTAA AATGTA GTAATA CTTTTG GAACTT GCAGGT CAGATT CTTTTA
17281 TAGGGG ACACAC TAAGGG AGCTTG GGTGAT AGTTGG TAAAAT GTGTTT CAAGTG ATGAAA
17341 ACTTGA ATTATT ATCACC GCAACC TACTTT TTAAAA AAAAAA GCCAGG CCTGTT AGAGCA
17401 TGCTTA AGGGAT CCCTAG GACTTG CTGAGC ACACAA GAGTAG TTACTT GGCAGG CTCCTG
17461 GTGAGA GCATAT TTCAAA AAACAA GGCAGA CAACCA AGAAAC TACAGT TAAGGT TACCTG
17521 TCTTTA AACCAT CTGCAT ATACAC AGGGAT ATTAAA ATATTC CAAATA ATATTT CATTCA
17581 AGTTTT CCCCCA TCAAAT TGGGAC ATGGAT TTCTCC GGTGAA TAGGCA GAGTTG GAAACT
17641 AAACAA ATGTTG GTTTTG TGATTT GTGAAA TTGTTT TCAAGT GATAGT TAAAGC CCATGA
17701 GATACA GAACAA AGCTGC TATTTC GAGGTC TCTTGG TTTATA CTCAGA AGCACT TCTTTG
17761 GGTTTC CCTGCA CTATCC TGATCA TGTGCT AGGCCT ACCTTA GGCTGA TTGTTG TTCAAA
17821 TAAACT TAAGTT TCCTGT CAGGTG ATGTCA TATGAT TTCATA TATCAA GGCAAA ACATGT
17881 TATATA TGTTAA ACATTT GTACTT AATGTG AAAGTT AGGTCT TTGTGG GTTTGA TTTTTA
17941 ATTTTC AAAACC TGAGCT AAATAA GTCATT TTTACA TGTCTT ACATTT GGTGGA ATTGTA
18001 TAATTG TGGTTT GCAGGC AAGACT CTCTGA CCTAGT AACCCT ACCTAT AGAGCA CTTTGC
18061 TGGGTC ACAAGT CTAGGA GTCAAG CATTTC ACCTTG AAGTTG AGACGT TTTGTT AGTGTA
18121 TACTAG TTTATA TGTTGG AGGACA TGTTTA TCCAGA AGATAT TCAGGA CTATTT TTGACT
18181 GGGCTA AGGAAT TGATTC TGATTA GCACTG TTAGTG AGCATT GAGTGG CCTTTA GGCTTG
18241 AATTGG AGTCAC TTGTAT ATCTCA AATAAT GCTGGC CTTTTT TAAAAA GCCCTT GTTCTT

18301 TATCAC CCTGTT TTCTAC ATAATT TTTGTT CAAAGA AATACT TGTTTG GATCTC CTTTTG

18361 ACAACA ATAGCA TGTTTT CAAGCC ATATTT TTTTTC CTTTTT TTTTTT TTTTTT GGTTTT

18421 TCGAGA CAGGGT TTCTCT GTATAG CCCTGG CTGTCC TGGAAC TCACTT TGTAGA CCAGGC

18481 TGGCCT CGAACT CAGAAA TCCGCC TGCCTC TGCCTC CTGAGT GCCGGG ATTAAA GGCGTG

18541 CACCAC CACGCC TGGCTA AGTTGG ATATTT TGTTAT ATAACT ATAACC AATACT AACTCC

18601 ACTGGG TGGATT TTTAAT TCAGTC AGTAGT CTTAAG TGGTCT TTATTG GCCCTT CATTAA

18661 AATCTA CTGTTC ACTCTA ACAGAG GCTGTT GGTACT AGTGGC ACTTAA GCAACT TCCTAC

18721 GGATAT ACTAGC AGATTA AGGGTC AGGGAT AGAAAC TAGTCT AGCGTT TTGTAT ACCTAC

18781 CAGCTT TATACT ACCTTG TTCTGA TAGAAA TATTTC AGGACA TCTAGA GTGTAC TATAAG

18841 GTTGAT GGTAAG CTTATA AGGAAC TTGAAA GTGGAG TAACTA CTCCAT TTCTCT GAGGGG

18901 AGAATT AAAATT TTTGAC CAAGTG TTGTTG AGCCAC TGAGAA TGGTCT CAGAAC ATAACT

18961 TCTTAA GGAACC TTCCCA GATTGC CCTCAA CACTGC ACCACA TTTGGT CCTGCT TGAACA

19021 TTGCCA TGGCTC TTAAAG TCTTAA TTAAGA ATATTA ATTGTG TAATTA TTGTTT TTCCTC

19081 CTTTAG ATCATT CCTTGA GGACAG GACAGT GCTTGT TTAAGG CTATAT TTCTGC TGTCTG

19141 AGCAGC AACAGG TCTTCG AGATCA ACATGA TGTTCA TAATCC CAAGAT GTTGCC ATTTAT

19201 GTTCTC AGAAGC AAGCAG AGGCAT GATGGT CAGTGA CAGTAA TGTCAC TGTGTT AAATGT

19261 TGCTAT GCAGTT TGGATT TTTCTA ATGTAG TGTAGG TAGAAC ATATGT GTTCTG TATGAA

19321 TTAAAC TCTTAA GTTACA CCTTGT ATAATC CATGCA ATGTGT TATGCA ATTACC ATTTTA

19381 AGTATT GTAGCT TTCTTT GTATGT GAGGAT AAAGGT GTTTGT CATAAA ATGTTT TGAACA

19441 TTTCCC CAAAGT TCCAAA TTATAA AACCAC AACGTT AGAACT TATTTA TGAACA ATGGTT

19501 GTAGTT TCATGC TTTTAA AATGCT TAATTA TTCAAT TAACAC CGTTTG TGTTAT AATATA

19561 TATAAA ACTGAC ATGTAG AAGTGT TTGTCC AGAACA TTTCTT AAATGT ATACTG TCTTTA

19621 GAGAGT TTAATA TAGCAT GTCTTT TGCAAC ATACTA ACTTTT GTGTTG GTGCGA GCAATA

19681 TTGTGT AGTCAT TTTGAA AGGAGT CATTTC AATGAG TGTCAG ATTGTT TTGAAT GTTATT

19741 GAACAT TTTAAA TGCAGA CTTGTT CGTGTT TTAGAA AGCAAA ACTGTC AGAAGC TTTGAA

19801 CTAGAA ATTAAA AAGCTG AAGTAT TTCAGA AGGGAA ATAAGC TACTTG CTGTAT TAGTTG

19861 AAGGAA AGTGTA ATAGCT TAGAAA ATTTAA AACCAT ATAGTT GTCATT GCTGAA TATCTG

19921 GCAGAT GAAAAG AAATAC TCAGTG GTTCTT TTGAGC AATATA ACAGCT TGTTAT ATTAAA

19981 AATTTT CCCCAC AGATAT AAACTC TAATCT ATAACT CATAAA TGTTAC AAATGG ATGAAG

20041 CTTACA AATGTG GCTTGA CTTGTC ACTGTG CTTGTT TTAGTT ATGTGA AAGTTT GGCAAT

20101 AAACCT ATGTCC TAAATA GTCAAA CTGTGG AATGAC TTTTTA ATCTAT TGGTTT GTCTAG

20161 AACAGT TATGTT GCCATT TGCCCT AATGGT GAAAGA AAAAGT GGGGAG TGCCTT GGCACT

20221 GTTCAT TTGTGG TGTGAA CCAAAG AGGGGG GCATGC ACTTAC ACTTCA AACATC CTTTTG

20281 AAAGAC TGACAA GTTTGG GTCTTC ACAGTT GGAATT GGGCAT CCCTTT TGTCAG GGAGGG

20341 AGGGAG GGAGGG AGGCTG GCTTGT TATGCT GACAAG TGTGAT TAAATT CAAACT TTGAGG

20401 TAAGTT GGAGGA ACTTGT ACATTG TTAGGA GTGTGA CAATTT GGACTC TTAATG ATTTGG

20461 TCATAC AAAATG AACCTA GACCAA CTTCTG GAAGAT GTATAT AATAAC TCCATG TTACAT

20521 TGATTT CACCTG ACTAAT ACTTAT CCCTTA TCAATT AAATAC AGAAGA TGCCAG CCATCT

20581 GGGCCT TTTAAC CCAGAA ATTTAG TTTCAA ACTCCT AGGTTA GTGTTC TCACTG AGCTAC

20641 ATCCTG ATCTAG TCCTGA AAATAG GACCAC CGGCGC GCCACT CTTCGC GACAGC TAGATC

20701 TCATCG CCTAGG ATCGCC CGGGTT GATTCG AGGCTG CTAACA AATCGA GCAGTG TGGTTT

20761 TCAAGA GGAAGC AAAAAG CCTCTC CACCCA GGCCTG GAATGT TTCCAC CCAATG TCGAGC

20821 AGTGTG GTTTTG CAAGAG GAAGCA AAAAGC CTCTCC ACCCAG GCCTGG AATGTT TCCACC

20881 CAATGT CGAGCA AACCCC GCCCAG CGTCTT GTCATT GGCGAA TTCGAA CACGCA GATGCA

20941 GTCGGG GCGGCG CGGTCC GAGGTC CACTTC GCATAT TAAGGT GACGCG TGTGGC CTCGAA

21001 CACCGA GCGACC CTGCAG CGACCC GCTTAA CAGCGT CAACAG CGTGCC GCAGAT CTTGGT

21061 GGCGTG AAACTC CCGCAC CTCTTC GGCCAG CGCCTT GTAGAA GCGCGT GCCATG GATCCT

21121 GATGAT GTTGTT GATTCT TCTAAA TCTTTT GTGATG GAAAAC TTTTCT TCGTAC CACGGG

21181 ACTAAA CCTGGT TATGTA GATTCC ATTCAA AAAGGT ATACAA AAGCCA AAATCT GGTACA

21241 CAAGGA AATTAT GACGAT GATTGG AAAGGG TTTTAT AGTACC GACAAT AAATAC GACGCT

21301 GCGGGA TACTCT GTAGAT AATGAA AACCCG CTCTCT GGAAAA GCTGGA GGCGTG GTCAAA

21361 GTGACG TATCCA GGACTG ACGAAG GTTCTC GCACTA AAAGTG GATAAT GCCGAA ACTATT

21421 AAGAAA GAGTTA GGTTTA AGTCTC ACTGAA CCGTTG ATGGAG CAAGTC GGAACG GAAGAG

21481 TTTATC AAAAGG TTCGGT GATGGT GCTTCG CGTGTA GTGCTC AGCCTT CCCTTC GCTGAG

21541 GGGAGT TCTAGC GTTGAA TATATT AATAAC TGGGAA CAGGCG AAAGCG TTAAGC GTAGAA

21601 CTTGAG ATTAAT TTTGAA ACCCGT GGAAAA CGTGGC CAAGAT GCGATG TATGAG TATATG

21661 GCTCAA GCCTGT GCAGGA AATCGT GTCAGG CGATCT CTTTGT GAAGGA ACCTTA CTTCTG

21721 TGGTGT GACATA ATTGGA CAAACT ACCTAC AGAGAT TTAAAG CTCTAA GGTAAA TATAAA

21781 ATTTTT AAGTGT ATAATG TGTTAA ACTACT GATTCT AATTGT TTGTGT ATTTTA GATTCC

21841 AACCTA TGGAAC TGATGA ATGGGA GCAGTG GTGGAA TGCAGA TCCTAG AGCTCG CTGATC

21901 AGCCTC GACTGT GCCTTC TAGTTG CCAGCC ATCTGT TGTTTG CCCCTC CCCCGT GCCTTC

21961 CTTGAC CCTGGA AGGTGC CACTCC CACTGT CCTTTC CTAATA AAATGA GGAAAT TGCATC

22021 GCATTG TCTGAG TAGGTG TCATTC TATTCT GGGGGG TGGGGT GGGGCA GGACAG CAAGGG

22081 GGAGGA TTGGGA AGACAA TAGCAG GCATGC TGGGGA TGCGGT GGGCTC TATGGC TTCTGA

22141 GGCGGA AAGAAC CAGCCC GGGCGG TGGAGC TCCAAT TCGCCC TATAGT GAGTCG TATTAC

22201 AATTCA CTGGCC GTCGTT TTACAA CGTCGT GACTGG GAAAAC CCTGGC GTTACC CAACTT

22261 AATCGC CTTGCA GCACAT CCCCCT TTCGCC AGCTGG CGTAAT AGCGAA GAGGCC CGCACC

22321 GATCGC CCTTCC CAACAG TTGCGC AGCCTG AATGGC GAATGG AAATTG TAAGCG

Notes: Homology arms; KI region; SDA sites; loxP sites; Exons; Sequence confirmed regions
