# Supplementary material for: Generation and Application of Inducible Chimeric RNA ASTN2-PAPPAas Knockin Mouse Model
Source: Cells. 2022 Jan 14;11(2):277. doi: 10.3390/cells11020277 (PMC8773765; doi:10.3390/cells11020277)
Supplement: Supplementary file 1 [file cells-11-00277-s001.zip › cells-1465623-supplementary/Supplementary Table S2.pdf]

Supplementary Table S2. The fertility of two groups of female mice (Mean ± SEM)

**Parameter**	**WT**<br>**（n = 3）**	***A*-*P*$_{as}$chiRNA KI**<br>**（n = 3）**
Average number of pregnancies per year	9.33±0.33	9.0±0.58
The average number of births per litter	7.80±0.20	8.00±0.20
Total breeding population per year	195	192
Survival rate of mice % (n)	94.36（184）	95.31（183）
