# Supplementary material for: Generation and Application of Inducible Chimeric RNA ASTN2-PAPPAas Knockin Mouse Model
Source: Cells. 2022 Jan 14;11(2):277. doi: 10.3390/cells11020277 (PMC8773765; doi:10.3390/cells11020277)
Supplement: Supplementary file 1 [file cells-11-00277-s001.zip › cells-1465623-supplementary/Supplementary Table S3.pdf]

Supplementary Table S3. Hematological counts of two groups of mice (Mean ± SEM)

**Parameter**	**WT**<br>**（n = 6）**	***A-P*$_{as}$chiRNA KI**<br>**（n = 6）**
WBC (10^9/L)	1.7±0.27	2.57±0.502
Lymphocytes (10^9/L)	1.32±0.26	2.17±0.47
Monocytes (10^9/L)	0.67±0.33	0.67±0.21
Neutrophils (10^9/L)	0.32±0.09	0.33±0.07
RBC (10^{12}/L)	7.10±0.59	7.80±0.28
HGB (g/L)	120.17±7.60	126.67±5.10
HCT (%)	34.28±3.36	40.27±1.95
PLT (10^9/L)	554.67±105.61	601.67±40.20
