# Supplementary material for: Generation and Application of Inducible Chimeric RNA ASTN2-PAPPAas Knockin Mouse Model
Source: Cells. 2022 Jan 14;11(2):277. doi: 10.3390/cells11020277 (PMC8773765; doi:10.3390/cells11020277)
Supplement: Supplementary file 1 [file cells-11-00277-s001.zip › cells-1465623-supplementary/Supplementary Table S4.pdf]

Supplementary Table S4. Serum biochemical indices of two groups of mice (Mean ± SEM)

**Parameter**	**WT** **（n = 6）**	***A-P*$_{as}$chiRNA KI** **（n = 6）**
ALB (g/L)	31.973±0.612	32.155±0.794
AST (U/L)	162.755±11.198	137.064±3.032
ALT (U/L)	112.672±4.648	100.891±3.021
TBA (umol/ L)	4.832±2.013	7.467±1.72
DBIL (umol/ L)	8.064±0.836	6.954±0.613
TBIL (umol/ L)	13.885±0.871	17.669±1.771
UA (umol/L)	116.067±16.897	112.758±4.88
CREA (umol/L)	31.079±5.43	19.667±2.93
BUN (mg/dL)	23.925±1.133	21.339±0.94
